# Modeling the cell-type-specific mesoscale murine connectome with anterograde tracing experiments

**DOI:** 10.1162/netn_a_00337

**Published:** 2023-12-22

**Authors:** Samson Koelle, Dana Mastrovito, Jennifer D. Whitesell, Karla E. Hirokawa, Hongkui Zeng, Marina Meila, Julie A. Harris, Stefan Mihalas

**Affiliations:** Allen Institute for Brain Science, Seattle, WA, USA; Department of Statistics, University of Washington, Seattle, WA, USA

**Keywords:** Connectivity, Cell type, Mouse

## Abstract

The Allen Mouse Brain Connectivity Atlas consists of anterograde tracing experiments targeting diverse structures and classes of projecting neurons. Beyond regional anterograde tracing done in C57BL/6 wild-type mice, a large fraction of experiments are performed using transgenic Cre-lines. This allows access to cell-class-specific whole-brain connectivity information, with class defined by the transgenic lines. However, even though the number of experiments is large, it does not come close to covering all existing cell classes in every area where they exist. Here, we study how much we can fill in these gaps and estimate the cell-class-specific connectivity function given the simplifying assumptions that nearby voxels have smoothly varying projections, but that these projection tensors can change sharply depending on the region and class of the projecting cells. This paper describes the conversion of Cre-line tracer experiments into class-specific connectivity matrices representing the connection strengths between source and target structures. We introduce and validate a novel statistical model for creation of connectivity matrices. We extend the Nadaraya-Watson kernel learning method that we previously used to fill in spatial gaps to also fill in gaps in cell-class connectivity information. To do this, we construct a “cell-class space” based on class-specific averaged regionalized projections and combine smoothing in 3D space as well as in this abstract space to share information between similar neuron classes. Using this method, we construct a set of connectivity matrices using multiple levels of resolution at which discontinuities in connectivity are assumed. We show that the connectivities obtained from this model display expected cell-type- and structure-specific connectivities. We also show that the wild-type connectivity matrix can be factored using a sparse set of factors, and analyze the informativeness of this latent variable model.

## Author Summary

While standard connectivity studies in mammalian models focus on the overall connection strength between areas, there is an increasing focus on specific connection types between cell classes when describing functions at a local circuit level. Having recently described the importance of such classes in the cortico-thalamic system, we now investigate their importance for estimating brain-wide mesoscopic connectivity. Even within our relatively large dataset, the connectivity data across cell classes is sparse, and so we introduce a method to more reliably extrapolate across classes and estimate connection-type-specific inter-areal connectivity. We observe that this complex connectivity may be described via a relatively small set of factors. While not complete, this connectivity matrix represents a categorical and quantitative improvement in mouse mesoscale connectivity models.

## INTRODUCTION

The mammalian nervous system enables an extraordinary range of natural behaviors and has inspired much of modern artificial intelligence. Neural connections including those from one region to another form the architecture underlying this capability. These **connectivities** vary by neuron type, as well as source (cell body) location and target (axonal projection) structures. Thus, characterization of the relationship between neuron type and source and target structure is important for understanding the overall nervous system.

Viral tracing experiments—in which a viral vector expressing green fluorescent protein (GFP) is transduced into neural cells through stereotaxic injection—are a useful tool for mapping these connections on the mesoscale (Chamberlin, Du, de Lacalle, & Saper, 1998; Daigle et al., 2018; J. A. Harris, Oh, & Zeng, 2012). The long-range connections between different areas are generally formed by axons that travel from one region to another, and the GFP protein moves into the axon of the projecting neurons. Two-photon tomography imaging can be used to determine the location and strength of the fluorescent signals in two-dimensional slices. These locations can then be mapped back into three-dimensional space, and the signal may then be integrated over area into cubic **voxels** to give a finely quantized three-dimensional fluorescence.

Several statistical models for the conversion of such experiment-specific signals into generalized estimates of connectivity strength have been proposed (Gămănuţ et al., 2018; K. D. Harris, Mihalas, & Shea-Brown, 2016; Knox et al., 2019; Oh et al., 2014). Of these, Oh et al. (2014) and Knox et al. (2019) provide a model for regionalized connectivities, which are voxel connectivities integrated by region. The value of these models is that they provide some improvement over simply averaging the projection signals of injections in a given region. However, these previous works model connectivities observed only in wild-type mice, which are suboptimally suited to assessment of cell-type-specific connectivity compared with fluorescence from Cre-recombinase-induced enhanced GFP (eGFP) expression in cell types specified by the combination of transgenic mouse strain and transgene promoter (J. A. Harris et al., 2019). We generally refer to sets of so-targeted eGFP-expressing cells in tracing experiments as a **cell class** since they may contain multiple types. For example, use of both wild-type and transgenic mice would give rise to cell-class-specific experiments, albeit with different yet perhaps overlapping classes of cells.

Thus, this paper introduces a class-specific statistical model for anterograde tracing experiments that synthesizes the diverse set of **Cre-lines** described in J. A. Harris et al. (2019), and expands this model to the entire mouse brain. Our model is, to our knowledge, a novel statistical estimator that takes into account both the spatial position of the labeled source, as well as the categorical cell class. Like the previously state-of-the-art model in Knox et al. (2019), this model predicts regionalized connectivity as an average over positions within the structure, with nearby experiments given more weight. However, our model weighs class-specific behavior in a particular structure against spatial position, so a nearby experiment specific to a similar cell class is relatively up-weighted, while a nearby experiment specific to a dissimilar class is down-weighted. This model outperforms the model of Knox et al. (2019) based on its ability to predict held-out experiments in leave-one-out cross-validation. We use the trained model to estimate overall connectivity matrices for each assayed cell class.

The resulting cell-class-specific connectivity is a directed weighted multigraph that can be represented as a **tensor** with missing values. We do not give an exhaustive analysis of these data, but do establish a lower limit of detection, verify several cell-type-specific connectivity patterns found elsewhere in the literature, and show that these cell-type-specific signals are behaving in expected ways. We also decompose the wild-type connectivity matrix into factors representing latent connectivity patterns, which we call archetypes. These components allow approximation of the regionalized connectivity using linear combinations of a small set of components.

The Methods section gives information on the data and statistical methodology, and the Results section presents our findings. These include connectivities, assessments of model fit, and subsequent biological and statistical analyses. Additional information on our dataset, methods, and results are given in Supporting Information Sections 5, 6, and 7, respectively.

## METHODS

We estimate and analyze cell-class-specific connectivity functions using models trained on murine brain viral tracing experiments. This section describes the data used to generate the model, the model itself, the evaluation of the model against its alternatives, and the use of the model in creation of the connectivity estimate matrices. It also includes background on the nonnegative matrix factorization method used for decomposing the wild-type connectivity matrix into latent factors. Additional information about our data and methods are given in Supporting Information Sections 5 and 6, respectively.

### Data

**Figure 1.**
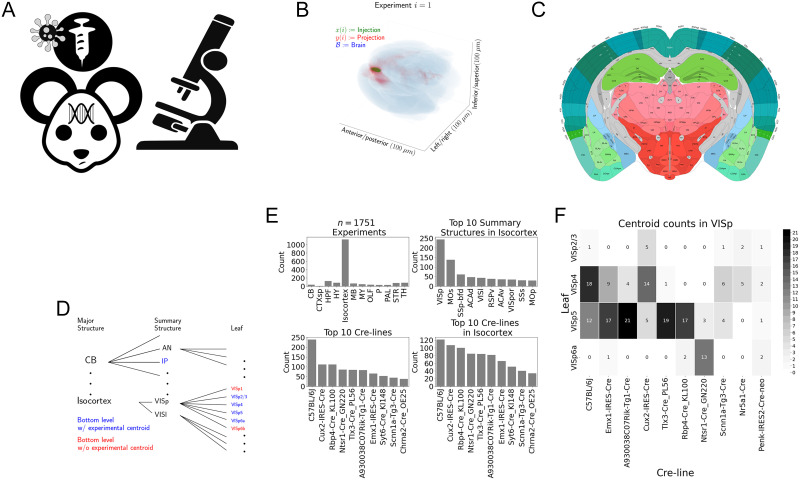
Experimental setting. (A) For each experiment, a Cre-dependent eGFP-expressing transgene casette is transduced by stereotaxic injection into a Cre-driver mouse, followed by serial two-photon tomography imaging. (B) An example of the segmentation of projection (targets) and injection (source) for a single experiment. Within each brain (blue), injection (green), and projection (red) areas are determined via histological analysis and alignment to the Allen Common Coordinate Framework (CCF). (C) Brain region parcellations within a coronal plane of CCFv3. (D) Explanation of nested structural ontology highlighting various levels of CCFv3 structure ontology. Lowest level (leaf) structures are colored in blue, and structures without an injection centroid are colored in red. (E) Abundances of tracer experiments by Cre-line and region of injection. (F) Co-occurrence of layer-specific centroids and Cre-lines within VISp.

Our dataset 𝒟 consists of *n* = 1,751 publicly available murine brain viral tracing experiments from the Allen Mouse Brain Connectivity Atlas. Figure 1A summarizes the experimental process used to generate these data. In each experiment, a mouse is injected with an adeno-associated virus (AAV) encoding GFP into a single location in the brain. Location of fluorescence is mediated by the location of the injection, the characteristics of the transgene, and the genotype of the mouse. In particular, Cre-driver or, equivalently, Cre-line mice are engineered to express Cre under the control of a specific and single gene promoter. This localizes expression of Cre to regions with certain transcriptomic cell-type signatures. In such Cre-driver mice, we used a double-inverted floxed AAV to produce eGFP fluorescence that depends on Cre expression in infected cells. To account for the complex cell-type targeting induced by a particular combination of Cre-driver genotype and GFP promoter, we refer to the combinations of cell types targeted by a particular combination of AAV and Cre-driver mice as cell classes. For example, we include experiments from Cre-driver lines that selectively label cell classes located in distinct cortical layers or other nuclei across the whole brain. For injections in the wild-type mice, we used the Synapsin I promoter (Jackson, Dayton, Deverman, & Klein, 2016; Kügler, Kilic, & Bähr, 2003). For injections into Cre mice, we used the CAG promoter with a Flex cassette for Cre-mediated recombination control (Saunders, Johnson, & Sabatini, 2012). Additional details are given in J. A. Harris et al. (2019).

For each experiment, the fluorescent signal imaged after injection is aligned into the Allen Common Coordinate Framework (CCF) v3, a three-dimensional average template brain that is fully annotated with regional parcellations (Wang et al., 2020). The whole-brain imaging and registration procedures described in detail in Kuan et al. (2015) and Oh et al. (2014) produce quantitative metrics of fluorescence discretized at the 100 *μ*m voxel level. Given an experiment, this image was histologically segmented by an analyst into *injection* and *projection* areas corresponding to areas containing somas, dendrites, and axons or exclusively axons of the transfected neurons. An example of a single experiment rendered in 3D is given in Figure 1B. Given an experiment *i*, we represent injections and projections as functions *x*(*i*), *y*(*i*) : ℬ → ℝ_≥0_, where ℬ ⊂ [1 : 132] × [1 : 80] × [1 : 104] corresponds to the subset of the (1.32 × 0.8 × 1.04) cm rectangular space occupied by the standard voxelized mouse brain. We also calculate injection centroids *c*(*i*) ∈ ℝ^3^ and regionalized projections *y*_𝒯_(*i*) ∈ ℝ^T^ given by the sum of *y*(*i*) in each region. A description of these steps is in Supporting Information Section 6.

Our goal is the estimation of regionalized connectivity from one region to another. A visual depiction of this region parcellation for a two-dimensional slice of the brain is given in Figure 1C. All structures annotated in the CCF belong to a hierarchically ordered ontology, with different areas of the brain parcellated to differing finer depths within a hierarchical tree. We denote the main levels of interest as major structures, summary structures, and layers. Not every summary structure has a layer decomposition within this ontology, so we typically consider the finest possible regionalization—for example, layer within the cortex, and summary structure within the thalamus, and denote these structures as leafs. As indicated in Figure 1D, the dataset used to generate the connectivity model reported in this paper contains certain combinations of region and cell class frequently, and others not at all. A summary of the most frequently assayed cell classes and structures is given in Figures 1E and 1F. Since users of the connectivity matrices may be interested in particular combinations, or interested in the amount of data used to generate a particular connectivity estimate, we present this information about all experiments in Supporting Information Section 5.

### Modeling Regionalized Connectivity

We define voxelized cell-class-specific connectivity *f* : 𝒱 × ℬ × → ℝ_≥0_ as giving the voxelized connectivity strength of a particular cell class from a source voxel to a target voxel. In contrast to Knox et al. (2019), which uses only wild-type C57BL/6J mice, our dataset has experiments targeting ∣𝒱∣ = 114 different combinations of Cre-driver mice and Cre-regulated AAV transgenes jointly denoted as 𝒱 ≔ {𝓋}. As in Knox et al. (2019), we ultimately estimate an integrated regionalized connectivity defined with respect to a set of *S* = 564 source leafs 𝒮 ≔ {*s*} and *T* = 1,123 target leafs 𝒯 ≔ {*t*}, of which 1,123 − 564 = 559 are contralateral. That is, we define

$$\begin{array}{l} \mathrm{regionalized}\;\mathrm{connectivity}\;\mathrm{strength}\;𝒞:𝒱\times 𝒮\times 𝒯\to {\mathbb{R}}_{\geq 0}\;\text{with}\;𝒞\left(v,s,t\right)=\sum_{l_{j}\in s}\sum_{l_{j^{\prime }}\in t}f\left(v,l_{j},l_{j^{\prime }}\right),\\ \mathrm{normalized}\;\mathrm{regionalized}\;\mathrm{connectivity}\;\mathrm{strength}\;{𝒞}^{N}:𝒱\times 𝒮\times 𝒯\to {\mathbb{R}}_{\geq 0}\;\text{with}\;{𝒞}^{N}\left(v,s,t\right)=\frac{1}{|s|}𝒞\left(v,l_{j},l_{j^{\prime }}\right),\\ \mathrm{normalized}\;\mathrm{regionalized}\;\mathrm{projection}\;\mathrm{density}\;{𝒞}^{D}:𝒱\times 𝒮\times 𝒯\to {\mathbb{R}}_{\geq 0}\;\text{with}\;{𝒞}^{D}\left(v,s,t\right)=\frac{1}{|s||t|}𝒞\left(v,l_{j},l_{j^{\prime }}\right), \end{array}$$

where *l*_*j*_ and *l*_*j*′_ are the locations of source and target voxels, and ∣*s*∣ and ∣*v*∣ are defined to be the number of voxels in the source and target structure, respectively. Since the normalized strength and densities are computable from the strength via a fixed normalization, our main statistical goal is to estimate 𝒞(*v*, *s*, *t*) for all *v*, *s*, and *t*. In other words, we want to estimate matrices 𝒞_*v*_ ∈ ${\mathbb{R}}_{\geq 0}^{S\times T}$. We call this estimator $\hat{𝒞}$.

Construction of such an estimator raises the questions of what data to use for estimating which connectivity, how to featurize the dataset, what statistical estimator to use, and how to reconstruct the connectivity using the chosen estimator. We represent these considerations as

$$\hat{𝒞}\left(v,s,t\right)=f^{*}(\hat{f}\left(f_{*}\left(𝒟\left(v,s\right)\right)\right).$$ (1)

This makes explicit the data featurization f_*_, statistical estimator $\hat{f}$, and any potential subsequent transformation f* such as summing over the source and target regions. Denoting 𝒟 as a function of *v* and *s* reflects that we consider using different data to estimate connectivities for different cell classes and source regions. Table 1 reviews estimators used for this data type used in previous work, as well as our two main extensions: the Cre-NW and **expected loss** (EL) models. The main differences in our data featurization from Knox et al. (2019) are that we regionalize our data at the leaf level where available so that its layer-specific behavior is visible, and normalize our data by projection signal in order to account for differences between cell class. Additional model selection results are given in Supporting Information Section 5 for alternative normalization strategies, and more detail on estimation is given in Supporting Information Section 6.

**Table 1.** Estimation of 𝒞 using connectivity data. The regionalization, estimation, and featurization steps are denoted by f*, $\hat{f}$, and f_*_, respectively. The training data used to fit the model are given by index set *I*. We denote experiments with centroids in particular major brain divisions and leafs as *I*_*m*_ and *I*_*l*_, respectively. Data *I*_*l*_/*I*_*m*_ means that, given a location *l*_*s*_ ∈ *s* ∈ *m*, the model $\hat{f}$ is trained on all of *I*_*m*_, but uses only *I*_*l*_ for prediction. The nonnegative least squares (NNLS) estimator fits a linear model that predicts regionalized projection signal *y*_𝒯_ as a function of regionalized injection signal *x*_𝒮_. Thus, the regionalization step for a region *s* is given by applying the learned matrix $\hat{f}$ to the *s*th indicator vector. In contrast, the Nadaraya-Watson (NW) model is a local smoothing model that generates a prediction for each voxel within the source structure that are then averaged to estimate the structure-specific connectivity.

Name	f*	$\hat{f}$	f_*_	𝒟(*v*, *s*)
NNLS (Oh et al., 2014)	$\hat{f}$ (1_*s*_)	nnls(X, Y)	*X* = *x*_𝒮_, *Y* = *y*_𝒯_	*I*_*m*_/*I*_*m*_
NW (Knox et al., 2019)	∑_*l*_*s*_∈*s*_ $\hat{f}$ (*l*_*s*_)	nw(X, Y)	*X* = *l*_*s*_, *Y* = *y*_𝒯_	*I*_*m*_/*I*_*m*_
Cre-NW	∑_*l*_*s*_∈*s*_ $\hat{f}$ (*l*_*s*_)	nw(X, Y)	*X* = *l*_*s*_, *Y* = *y*_𝒯_	(*I*_*l*_ ∩ *I*_*v*_)/*I*_*m*_
Expected loss (EL)	∑_*l*_*s*_∈*s*_ $\hat{f}$ (*s*)	el(*X*, *Y*, *v*)	*X* = *l*_*s*_, *Y* = *y*_𝒯_, *v*	*I*_*l*_/*I*_*m*_

Our contributions—the Cre-NW and EL models—have several differences from the previous methods. In contrast to the nonnegative least squares (Oh et al., 2014) and **Nadaraya-Watson** (NW) (Knox et al., 2019) estimators that account only for source region *s*, our new estimators account for cell class *v*; The Cre-NW estimator uses experiments from only a particular class to predict connectivity for that class, while the EL estimator shares information between classes within a structure. Both of these estimators take into account both the cell class and the centroid position of the experimental injection. Like the NW and Cre-NW estimator, the EL estimator generates predictions for each voxel in a structure, and then sums them together to get the overall connectivity. However, in contrast to the NW approaches, the EL estimate of the projection vector for a cell class at a location weights the average projection of that cell class in the region containing the location against the relative locations of all experimental centroids in the region regardless of class. That is, cell class and source region combinations with similar average projection vectors will be up-weighted when estimating $\hat{f}$. Thus, all experiments that are nearby in three-dimensional space can help generate the prediction, even when there are few nearby experiments for the cell class in question. A detailed mathematical description of our new estimator is given in Supporting Information Section 6.

### Model Evaluation

We select optimum functions from within and between our estimator classes using leave-one-out cross-validation, in which the accuracy of the model is assessed by its ability to predict projection vectors experiments excluded from the training data on the basis of their cell class and experimental centroid. Equation 1 includes a deterministic step f* included without input by the data. The performance of $\hat{𝒞}$ (*v*, *s*, *t*) is thus determined by performance of $\hat{f}$(f_*_(𝒟(*v*, *s*))). Thus, we evaluate prediction of *f*_𝒯_ : ℝ^3^ → ${\mathbb{R}}_{\geq 0}^{T}$—the regionalized connection strength at a given location.

Another question is what combinations of *v*, *s*, and *t* to generate a prediction for. Our EL and Cre-NW models are leaf specific. They generate predictions only for cell classes in leafs where at least one experiment with a Cre-line targeting that class has a centroid. To accurately compare our new estimators with less restrictive models such as those used in Knox et al. (2019), we restrict our evaluation set to Cre-driver/leaf combinations that are present at least twice. The sizes of these evaluation sets are given in Supporting Information Section 5.

We use weighted *l*2-loss to evaluate these predictions.

$$\begin{array}{l} \text{l}2-\text{loss}\;\ell \left(y_{𝒯}\left(i\right)\right),\hat{y_{𝒯}\left(i\right))})≔∥y_{𝒯}\left(i\right))-\hat{y_{𝒯}\left(i\right))}{∥}_{2}^{2}.\\ \text{weighted}\;\text{l}2-\text{loss}\;ℒ\left(\hat{f\left(f_{*}\right)}\right)≔\frac{1}{|\left\{𝒮,𝒱\right\}|}\sum_{s,v\in \left\{𝒮,𝒱\right\}}\;\frac{1}{|I_{s}\cap I_{v}|}\sum_{i\in \left(I_{s}\cap I_{v}\right)}\;\ell \left(y_{𝒯}\left(i\right)\right),{\hat{f}}_{𝒯}\left(f_{*}\left(𝒟\left(v,s\right)\setminus i\right)\right). \end{array}$$

*I*_*s*_ refers to the set of experiments with centroid in structure *s*, and *I*_*v*_ refers to the set of experiments with Cre-line *v*, so ∣*I*_*s*_ ∩ *I*_*v*_∣ is the number of experiments of Cre-line *v* with injection centroid in structure *s*. This is a somewhat different loss from Knox et al. (2019) because of the increased weighting of rarer combinations of *s* and *v* implicit in the $\frac{1}{|I_{s}\cap I_{v}|}$ term in the loss. The establishment of a lower limit of detection and the extra estimation step used in the EL model to establish the relative importance of regionally averaged cell-class projection and injection centroid position are covered in Supporting Information Section 6.

### Connectivity Analyses

We examine our connectome estimates with both comparisons to known biology and statistical decompositions. As an exploratory analysis, we use heirarchical clustering to compare outputs from connectivities from different Cre-lines. Details of and results from this approach are given in Supporting Information Section 7. We then use nonnegative matrix factorization (NMF) to factor the wild-type connectivity matrix into a small set of underlying components that can be linearly combined to reproduce the observed long-range wild-type connectivity. Inspired by Mohammadi, Ravindra, Gleich, and Grama (2018), we refer to these latent coordinates as **connectivity archetypes** since they represent underlying patterns from which we can reconstruct a broad range of observed connectivities, although we note that the genomic archetypal analysis in that paper is slightly methodologically distinct.

NMF refers to a collection of **dictionary-learning** algorithms for decomposing a nonnegatively valued matrix such as 𝒞 into positively valued matrices called, by convention, weights *W* ∈ ${\mathbb{R}}_{\geq 0}^{S\times q}$ and hidden units *H* ∈ ${\mathbb{R}}_{\geq 0}^{q\times T}$. NMF assumes a simple linear statistical model: that the observed matrix is composed of linear combinations of latent coordinates (Devarajan, 2008). Unlike PCA (principal component analysis), NMF specifically accounts for the fact that data are all in the positive orthant, and it is more stable and interpretable in assays of complex biological systems than heirarchical clustering (Brunet, Tamayo, Golub, & Mesirov, 2004) The choice of matrix factorization method reflects particular scientific subquestions and probabilistic interpretations, and the matrix *H* may used to identify latent structures with interpretable biological meaning.

Our application of NMF to decompose the estimated long-range connectivity is of some independent interest, since we ignore connections between source and target regions less than 1,500 *μm* apart. This is because short-range projections resulting from diffusion and traveling fibers dominate the matrices $\hat{𝒞}$. Our NMF algorithm solves the following optimization problem:

$$\text{NMF}\left(𝒞,\lambda ,q\right)≔\arg\min_{W\in {\mathbb{R}}_{\geq 0}^{S\times q},H\in {\mathbb{R}}_{\geq 0}^{q\times T}}\frac{1}{2}∥1_{d\left(s,t\right)>1500\mu m}⊙𝒞-WH{∥}_{2}^{2}+\lambda \left(∥H{∥}_{1}+∥W{∥}_{1}\right).$$

We set *λ* = 0.002 to encourage sparser and therefore more interpretable components. We use unsupervised cross-validation to determine an optimum *q*, and show the top 15 stable components (Perry, 2009). Since the NMF objective is difficult to optimize and sensitive to initialization, we follow up with a stability analysis via clustering the resultant *H* from multiple replicates. The medians of the component clusters appearing frequently across NMF replicates are selected as connectivity archetypes. Details of these approaches are given in Sections 6 and 7 in the Supporting Information.

## RESULTS

The main result of this paper is the creation of cell-type-specific connectivity estimates from the Allen Mouse Brain Connectivity Atlas (MCA) experiments. We first establish that our new expected loss (EL) estimator performs best in validation assays for estimating wild-type and cell-type-specific connectivities. We then show that Cre-specific connectivity matrices generated using this model are consistent with known biology. Finally, we factor some of these connectivity matrices to show how connectivity arises from latent components, and that these latent components may be associated with cell types.

### Model Evaluation

Our EL model generally performs better than the other estimators that we consider. Table 2A contains weighted losses from leave-one-out cross-validation of candidate models, such as the NW Major-WT model from Knox et al. (2019). The EL model combines the good performance of class-specific models like NW Leaf-Cre in regions like isocortex with the good performance of class-agnostic models in regions like thalamus. Additional information on model evaluation, including class and structure specific performance, is given in Supporting Information Section 5. In particular, Supplementary Table 4 contains the sizes of these evaluation sets in each major structure, and Supporting Information Section 7 contains the structure- and class-specific losses.

**Table 2.**
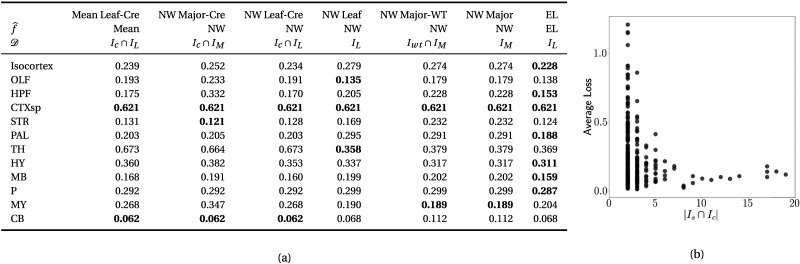
(A) Losses from leave-one-out cross-validation of candidate models. **Bold** numbers are best for their major structure. (B) Empirical performance of selected expected loss (EL) model by data abundance. The model is more accurate in Cre-leaf combinations where it draws on more data. The dataset variable 𝒟 indicates the set of experiments used to model a given connectivity. For example, *I*_*c*_ ∩ *I*_*L*_ means only experiments with a given Cre-line in a given leaf are used to model connectivity for the corresponding cell class in that leaf, while *I*_*L*_ means that all experiments in that leaf are used. *I*_*wt*_ ∩ *I*_*M*_ means all wild-type experiments in the major structure are used; this was the model in Knox et al. (2019).

### Connectivities

We estimate matrices ${\hat{𝒞}}_{v}\in {\mathbb{R}}_{\geq 0}^{S\times T}$ representing connections of source structures to target structures for particular Cre-lines *v*. We confirm the detection of several well-established connectivities within our tensor, although we expect additional interesting biological processes to be identifiable. The connectivity tensor and code to reproduce it are available at https://github.com/AllenInstitute/mouse_connectivity_models/tree/class-specific.

#### Overall connectivity.

**Figure 2.**
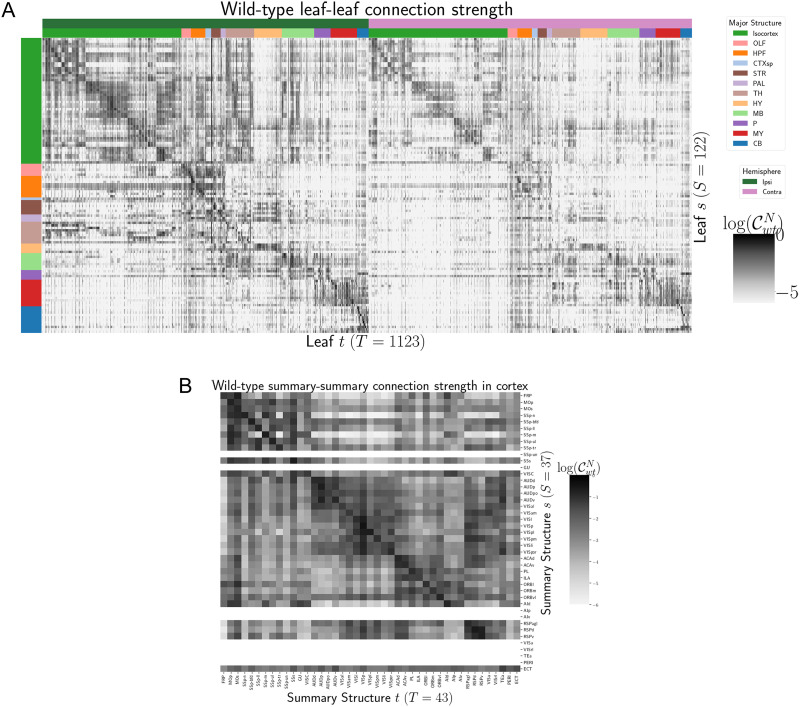
Wild-type connectivities. (A) Log wild-type leaf-to-leaf connectivity matrix log 𝒞(*s*, *t*, *v*_*wt*_). Sources and target major brain division structure are shown. (B) Log wild-type intracortical connectivity matrix at the summary structure level. Summary structures without an injection centroid are left blank.

Several known biological projection patterns are evident in the wild-type connectivity matrix 𝒞_*wt*_ shown in Figure 2A. This matrix shows connectivity from leaf sources to leaf targets. Large-scale patterns like intra-areal connectivities and ipsilateral connections between cortex and thalamus are clear, as in previous estimates in J. A. Harris et al. (2019), Knox et al. (2019), and Oh et al. (2014). However, the layer-specific targeting of the different Cre-lines enables our estimated wild-type connectivities to display heterogeneity at the layer level. This contrasts with the model in Knox et al. (2019), which is denominated as the NW Major-WT model, whose accuracy is evaluated in Table 2A. For comparison, we also plot averages over component layers weighted by layer size projections between summary-structure sources and targets in the cortex in Figure 2B. Importantly, as shown in Table 2A, this finer spatial resolution corresponds to the increased accuracy of our EL model over the NW Major-WT model.

#### Class-specific connectivities.

We investigate the presence of known biological processes within our connectivity estimates and confirm that these class-specific connectivities exhibit certain known behaviors. Although there is a rich anatomical literature using anterograde tracing data to describe projection patterns from subcortical sources to a small set of targets of interest, much of the accessible whole-brain projection data are from the MCA project used here to generate the connectome models. Thus, we compare with external studies to validate our results while avoiding a circular validation of the data used to generate the model weights. The cell types and source areas with extensive previous anatomical descriptions of projections using both bulk tracer methods with cell-type specificity and single-cell reconstructions that we investigate are (a) thalamic-projecting neurons in the visual and motor cortical regions; (b) cholinergic neurons in the medial septum and nucleus of the diagonal band (MS/NDB); and (c) serotonergic neurons of the dorsal raphe nucleus (DR). Our estimated connections are in agreement with literature on these cell types.

##### *Dependence of thalamic connection on cortical layer*.

**Figure 3.**
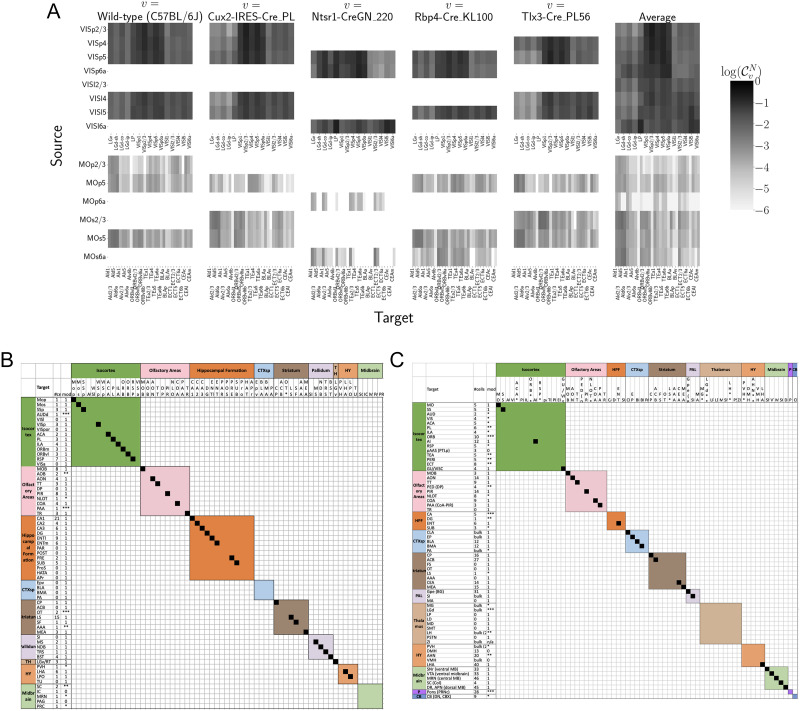
Cell-class specificity. (A) Selected cell-class- and layer-specific connectivities from two visual and two motor areas. Sources without an injection of a Cre-line are not estimated owing to lack of data. (B) Targets reported for cholinergic cells in MS/NDB in Li et al. (2018) and above the 90th percentile in our Chat-IRES-Cre-neo model. A black box along the diagonal indicates that the target was identified in both studies (*n* = 35). The number of single cells (out of 50) with a projection to each target is shown in the first column after the target acronym, and the next column shows whether a target appeared in the 90% thresholded model weights (1 = present, 0 = absent). Some of these targets appeared only at lower thresholds as indicated by asterisks: *** > 85th%, ** > 75th%, * > 50th%. (C) Targets reported for serotonergic cells in DR in Ren et al. (2018, 2019) and above the 90th percentile in our Slc6a4-Cre_ET33 model.

Visual cortical areas VISp and VISl and cortical motor areas MOp and MOs have established layer-specific projection patterns that can be labeled with the layer-specific Cre-lines from the Allen datasets and others (J. A. Harris et al., 2019; Jeong et al., 2016). Figure 3A shows that in VISp, the Ntsr1-Cre-line strongly targets the core part of the thalamic LGd nucleus, while in VISl, it is a strong projector to the LP nucleus. In VISp, the Rbp4-Cre-line strongly targets LP as well. Rbp4-Cre and Ntsr1-Cre injections target layers 5 and 6, respectively. Since we generate connectivity estimates only for structures with at least one injection centroid, this is shown by the position of nonzero rows in Figure 3A. To fill these gaps, as a heuristic alternative model, we also display an average connectivity matrix over all Cre-lines.

##### *MS and NDB projections in the Chat-IRES-Cre-neo model*.

Cholinergic neurons in the MS and NDB are well known to strongly innervate the hippocampus, olfactory bulb, piriform cortex, entorhinal cortex, and lateral hypothalamus (Watson, Paxinos, & Puelles, 2012; Zaborszky et al., 2015). In the Allen MCA, cholinergic neurons were labeled by injections into Chat-IRES-Cre-neo mice. Figure 3B checks the estimated connectome weights to targets in these major brain divisions from MS and NDB. We observed that all these expected divisions were represented above the 90th percentile of weights from these source structures.

We also compared our Chat-IRES-Cre connectome model data for MS and NDB with the targets identified by Li et al. (2018). This single-cell whole-brain mapping project using Chat-Cre mice fully reconstructed *n* = 50 cells to reveal these same major targets and also naming additional targets from MS/NDB (Li et al. 2018). We identified 150 targets at the fine leaf structure level among the top decile of estimated weights. To directly compare our data across studies, we merged structures as needed to get to the same ontology level, and removed ipsilateral and contralateral information. After formatting our data, we found 51 targets in the top 10%; Li et al. (2018) reported 47 targets across the 50 cells. There was good consistency overall between the target sets; 35 targets were shared, 12 were unique to the single-cell dataset, and 16 were unique to our model data. We checked whether targets were missing from our dataset to the threshold level. Indeed, lowering the threshold to the 75th percentile confirmed six more targets-in-common, and all but two targets from Li et al. (2018) were above the 50th percentile weights in our model. Of note, the absence of a target in the single-cell dataset that was identified in our model data is most likely due to the sparse sampling of all possible projections from only *n* = 50 MS/NDB cells.

##### *DR projections in the Slc6a4-Cre_ET33 model*.

Serotonergic projections from cells in the dorsal raphe (DR) are widely distributed and innervate many forebrain structures including isocortex and amygdala. In the Allen MCA, serotonergic neurons were labeled using Slc6a4-Cre_ET33 and Slc6a4-CreERT2_EZ13 mice. This small nucleus appears to contain a complex mix of molecularly distinct serotonergic neuron subtypes with some hints of subtype-specific projection patterns (Huang et al., 2019; Ren et al., 2018, 2019). We expect that the Cre-lines used here in the Allen MCA, which use the serotonin transporter promoter (Slc6a4-Cre and -CreERT2), will lead to expression of tracer in all the serotonergic subtypes recently described in an unbiased way, but this assumption has not been tested directly. We compared our model data with a single-cell reconstruction dataset consisting of *n* = 50 serotonergic cells with somas in the DR that also had bulk tracer validation (see Figure 3C). After processing our data to match the target structure ontology level across studies, we identified 37 targets from the DR with weights above the 90th percentile, whereas Ren et al. (2019) listed 55 targets across the single-cell reconstructions. Twenty-seven of these targets where shared.

Overall there was good consistency between targets in olfactory areas, cortical subplate, CP, ACB, and amygdala areas, as well in palidum and midbrain, while the two major brain divisions with the least number of matches are the isocortex and thalamus. There are a few likely reasons for these observations. First, in the isocortex, there is known to be significant variation in the density of projections across different locations, with the strongest innervation in lateral and frontal orbital cortices (Ren et al., 2019). Indeed, when we lower the threshold and check for weights of the targets outside of the 90%, we see all but one of these regions (PTLp, parietal cortex, which is not frontal or lateral) has a weight assigned in the top half of all targets. In the thalamus, our model predicted strong connections to several medial thalamic nuclei (i.e., MD, SMT) that were not targeted by the single cells. This discrepancy may be at least partially explained by the complex topographical organization of the DR that, like the molecular subtypes, is not yet completely understood. A previous bulk tracer study that specifically targeted injections to the central, lateral wings, and dorsal subregions of the DR reported semi-quantitative differences in projection patterns (Muzerelle, Scotto-Lomassese, Bernard, Soiza-Reilly, & Gaspar, 2016). Notably, Muzerelle et al. (2016) report that cells in the ventral region of DR project more strongly to medial thalamic nuclei, whereas the lateral and dorsal DR cells innervate more lateral regions (e.g., LGd). Thus, it is possible that the single-cell somas did not adequately sample the entire DR.

### Connectivity Analyses

While the manual analysis in the Results section is valuable for validation, scaling our interpretation of our connectivity estimates motivated us to apply dimension-reduction methods to our connectivity estimates and understand whether the learned structure agrees with the expected biology. For example, Supporting Information Section 7 shows a collection of connectivity strengths generated using Cre-specific models for wild-type, Cux2, Ntsr1, Rbp4, and Tlx3 Cre-lines from VIS areas at leaf level in the cortex to cortical and thalamic nuclei. Sorting source and target structure/cell-class combinations hierarchical clustering shows, for example, that layer 6–specific Ntsr1 Cre-lines source regions cluster together. This makes sense, since layer 6–specific Ntsr1 Cre-line distinctly projects to thalamic nuclei, regardless of source summary structure, and in contrast with the tendency of other cell classes to project to nearby regions within the cortex.

**Figure 4.**
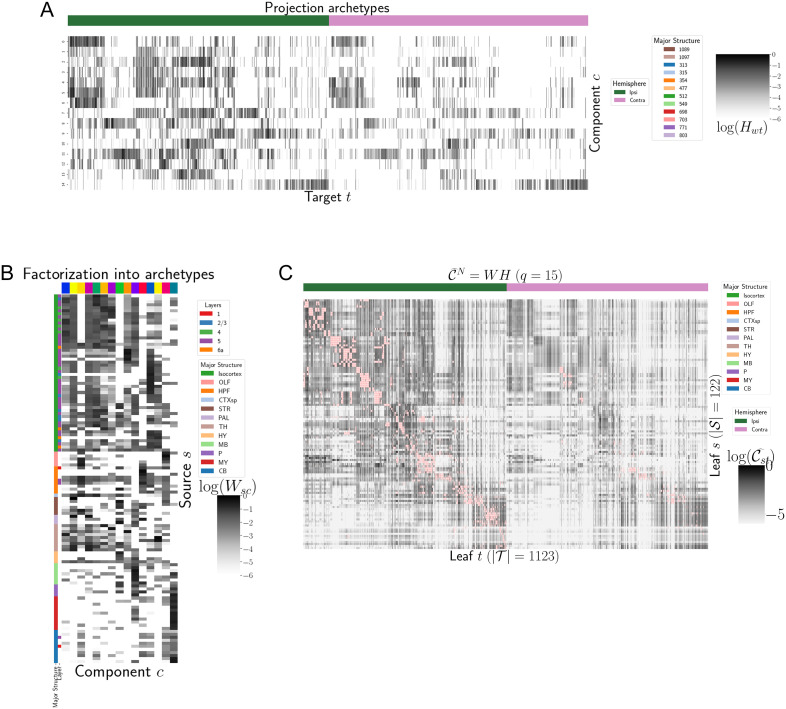
Nonnegative matrix factorization results ${𝒞}_{wt}^{N}$ = *WH* for *q* = 15 components. (A) Latent space coordinates *H* of 𝒞. Target major structure and hemisphere are plotted. (B) Loading matrix *W*. Source major structure and layer are plotted. (C) Reconstruction of the normalized distal connectivity strength using the top 15 archetypes. Areas less than 1,500 *μm* apart are not modeled, and therefore shown in pink.

In contrast with heirarchical clustering, nonnegative matrix factorization provides a simpler linear-model-based factorization (Hastie, Tibshirani, & Friedman, 2009). The low-dimensional coordinates returned by NMF nevertheless highlight important features within the connectivity matrix in a data-driven way. The learned projection archetypes *H* and model weights *W* are plotted in Figure 4. Intrastructural connections such as MB-MB MY-MY are visible in the 7th and 11th archetypes, but there is no obvious layer 6–specific signal. These factors may be used to generate a reconstructed connectivity matrix using the implied statistical model. Despite the relatively small number of learned additive factors, comparing with Figure 2A shows that this reconstructed matrix has a relatively globally plausible structure. Supporting Information Sections 6 and 7 contain quantitative performance of this model across choices of *q*, and assessment of factorization stability to ensure the decomposition is reliable across computational replicates. The Supporting Information also quantitatively shows differential association of projection archetypes in this model with projection vectors of sources from the Cux2, Ntsr1, Rbp4, and Tlx3 Cre-lines.

## DISCUSSION

The model presented here is among the first cell-type-specific whole-brain projectome models for a mammalian species, and it opens the door for a large number of models linking brain structure to computational architectures. Overall, we find expected targets, based on our anatomical expertise and published reports, but underscore that the core utility of this bulk connectivity analysis is not only in validation of existing connection patterns, but also in identification of new ones. We note that although the concordance appeared stronger for the cholinergic cells than the serotonergic cells, any differences might still be explained by the lack of high-quality “ground-truth” datasets to validate these Cre-line connectome models. It is important to note several limitations of the current analyses. Short-range connections can be affected by saturating signals near injection sites, as well as the segmentation algorithm capturing dendrites as well as axons. Furthermore, larger numbers of single-cell reconstructions that saturate all possible projection types would be a better gold standard than the small number of cells reported here. Future iterations of connectome models may also take into account single-cell axon projection data, or synthesize with retrograde tracing experiments.

The Nadaraya-Watson estimator using the cell-type space based on similarities of projections, and theoretical justification of the use of an intermediate **shape-constrained estimator**, provides an empirically useful new tool for categorical modeling. Ours is not the first cross-validation-based model averaging method (Gao, Zhang, Wang, & Zou, 2016), but our use of a shape-constrained estimator in target-encoded feature space is novel and fundamentally different from Nadaraya-Watson estimators that use an optimization method for selecting the weights (Saul & Roweis, 2003). The properties of this estimator and its relation to estimator fit using an optimization algorithm are therefore a possible future avenue of research (Groeneboom & Jongbloed, 2018; Salha & El Shekh Ahmed, 2015). Since in truth the impact of the viral tracer likely depends on the injection location in a nonlinear way, a deep-learning model could be appropriate, provided enough data were available, but our sample size seems too low to utilize a fixed-effect generative model (Lotfollahi, Naghipourfar, Theis, & Alexander Wolf, 2020). In a sense both the nonnegative least squares (Oh et al., 2014) and NW models can be thought of as improvements over the structure-specific average, and so it is also possible that a yet undeveloped residual-based data-driven blend of these models could provide improved performance. Finally, we note that a Wasserstein-based measure of injection similarity per structure could naturally combine the physical simplicity of the centroid model while also incorporating the full distribution of the injection signal.

The factorization of the connectivity matrix could also be improved. Nonlinear data transformations or matrix decompositions, or tensor factorizations that account for correlations between cell types, could better capture the true nature of latent neural connections (K. D. Harris et al., 2016). Cre- or layer-specific signal recovery as performed here could be used to evaluate a range of matrix decompositions. This could help for example to understand the influence of traveling fibers on the observed connectivity (Llano & Sherman, 2008). From a statistical perspective, a stability-based method for establishing archetypal connectivities in NMF is similar to those applied to genomic data (Kotliar et al., 2019; Wu et al., 2016). Regardless of statistical approach, as in genomics, latent low-dimensional organization in connectivity should inspire search for similarly parsimonious biological correlates.

## ACKNOWLEDGMENTS

We thank the Allen Institute for Brain Science founder, Paul G. Allen, for his vision, encouragement, and support.

## SUPPORTING INFORMATION

Supporting information for this article is available at https://doi.org/10.1162/netn_a_00337.

## AUTHOR CONTRIBUTIONS

Samson Koelle: Conceptualization; Formal analysis; Investigation; Software; Visualization; Writing – original draft. Dana Mastrovito: Formal analysis; Software; Validation. Jennifer D. Whitesell: Data curation; Investigation; Resources; Validation; Writing – review & editing. Karla E. Hirokawa: Data curation. Hongkui Zeng: Conceptualization; Funding acquisition; Supervision. Marina Meila: Formal analysis; Methodology; Supervision; Writing – review & editing. Julie A. Harris: Conceptualization; Data curation; Investigation; Supervision; Validation; Writing – review & editing. Stefan Mihalas: Conceptualization; Formal analysis; Investigation; Methodology; Supervision; Writing – review & editing.

## FUNDING INFORMATION

Stefan Mihalas, National Institute of Biomedical Imaging and Bioengineering (https://dx.doi.org/10.13039/100000070), Award ID: DA055669. Stefan Mihalas, National Institute of Biomedical Imaging and Bioengineering (https://dx.doi.org/10.13039/100000070), Award ID: EB029813.

## TECHNICAL TERMS

Structural connectivities: The matrix of connectivities between structures.
Voxel: A 100 *μm* cube of brain.
Cell class: The projecting neurons targeted by a particular Cre-line.
Cre-line: The combination of Cre-recombinase expression in transgenic mouse and Cre-induced expression in the vector that induces labeling of projection.
Structural connection tensor: Connectivities between structures across cell classes.
Expected loss: Our new estimator that weights location against cell class by their estimated predictive power.
Nadaraya-Watson: A simple local smoothing estimator with one parameter.
Connectivity archetypes: Projection patterns from which we can linearly reconstruct the connectome.
Dictionary-learning: A family of algorithms for finding low-dimensional data representations.
Shape constrained estimator: A statistical estimator that fits a function of a particular shape (e.g., monotonic increasing, convex).

## References

[bib1] Brunet, J.-P., Tamayo, P., Golub, T. R., & Mesirov, J. P. (2004). Metagenes and molecular pattern discovery using matrix factorization. Proceedings of the National Academy of Sciences, 101(12), 4164–4169. 10.1073/pnas.0308531101, 15016911 PMC384712

[bib2] Chamberlin, N. L., Du, B., de Lacalle, S., & Saper, C. B. (1998). Recombinant adeno-associated virus vector: Use for transgene expression and anterograde tract tracing in the CNS. Brain Research, 793(1–2), 169–175. 10.1016/S0006-8993(98)00169-3, 9630611 PMC4961038

[bib3] Daigle, T. L., Madisen, L., Hage, T. A., Valley, M. T., Knoblich, U., Larsen, R. S., … Zeng, H. (2018). A suite of transgenic driver and reporter mouse lines with enhanced brain-cell-type targeting and functionality. Cell, 174(2), 465–480. 10.1016/j.cell.2018.06.035, 30007418 PMC6086366

[bib4] Devarajan, K. (2008). Nonnegative matrix factorization: An analytical and interpretive tool in computational biology. PLoS Computational Biology, 4(7), e1000029. 10.1371/journal.pcbi.1000029, 18654623 PMC2447881

[bib5] Gămănuţ, R., Kennedy, H., Toroczkai, Z., Ercsey-Ravasz, M., Van Essen, D. C., Knoblauch, K., & Burkhalter, A. (2018). The mouse cortical connectome, characterized by an ultra-dense cortical graph, maintains specificity by distinct connectivity profiles. Neuron, 97(3), 698–715. 10.1016/j.neuron.2017.12.037, 29420935 PMC5958229

[bib6] Gao, Y., Zhang, X., Wang, S., & Zou, G. (2016). Model averaging based on leave-subject-out cross-validation. Journal of Econometrics, 192(1), 139–151. 10.1016/j.jeconom.2015.07.006

[bib7] Groeneboom, P., & Jongbloed, G. (2018). Some developments in the theory of shape constrained inference. Statistical Science, 33(4), 473–492. 10.1214/18-STS657

[bib8] Harris, J. A., Mihalas, S., Hirokawa, K. E., Whitesell, J. D., Choi, H., Bernard, A., … Zeng, H. (2019). Hierarchical organization of cortical and thalamic connectivity. Nature, 575(7781), 195–202. 10.1038/s41586-019-1716-z, 31666704 PMC8433044

[bib9] Harris, J. A., Oh, S. W., & Zeng, H. (2012). Adeno-associated viral vectors for anterograde axonal tracing with fluorescent proteins in nontransgenic and Cre driver mice. Current Protocols in Neuroscience, 59(1), 1.20.1–1.20.18. 10.1002/0471142301.ns0120s59, 22470147

[bib10] Harris, K. D., Mihalas, S., & Shea-Brown, E. (2016). Nonnegative spline regression of incomplete tracing data reveals high resolution neural connectivity.

[bib11] Hastie, T., Tibshirani, R., & Friedman, J. (2009). The elements of statistical learning. New York: Springer. 10.1007/978-0-387-84858-7

[bib12] Huang, K. W., Ochandarena, N. E., Philson, A. C., Hyun, M., Birnbaum, J. E., Cicconet, M., & Sabatini, B. L. (2019). Molecular and anatomical organization of the dorsal raphe nucleus. eLife, 8, e46464. 10.7554/eLife.46464, 31411560 PMC6726424

[bib13] Jackson, K. L., Dayton, R. D., Deverman, B. E., & Klein, R. L. (2016). Better targeting, better efficiency for wide-scale neuronal transduction with the synapsin promoter and AAV-PHP.B. Frontiers in Molecular Neuroscience, 9, 116. 10.3389/fnmol.2016.00116, 27867348 PMC5095393

[bib14] Jeong, M., Kim, Y., Kim, J., Ferrante, D. D., Mitra, P. P., Osten, P., & Kim, D. (2016). Comparative three-dimensional connectome map of motor cortical projections in the mouse brain. Scientific Reports, 6, 20072. 10.1038/srep20072, 26830143 PMC4735720

[bib15] Knox, J. E., Harris, K. D., Graddis, N., Whitesell, J. D., Zeng, H., Harris, J. A., … Mihalas, S. (2019). High-resolution data-driven model of the mouse connectome. Network Neuroscience, 3(1), 217–236. 10.1162/netn_a_00066, 30793081 PMC6372022

[bib16] Kotliar, D., Veres, A., Nagy, M. A., Tabrizi, S., Hodis, E., Melton, D. A., & Sabeti, P. C. (2019). Identifying gene expression programs of cell-type identity and cellular activity with single-cell RNA-Seq. eLife, 8, e43803. 10.7554/eLife.43803, 31282856 PMC6639075

[bib17] Kuan, L., Li, Y., Lau, C., Feng, D., Bernard, A., Sunkin, S. M., … Ng, L. (2015). Neuroinformatics of the Allen Mouse Brain Connectivity Atlas. Methods, 73, 4–17. 10.1016/j.ymeth.2014.12.013, 25536338

[bib18] Kügler, S., Kilic, E., & Bähr, M. (2003). Human synapsin 1 gene promoter confers highly neuron-specific long-term transgene expression from an adenoviral vector in the adult rat brain depending on the transduced area. Gene Therapy, 10(4), 337–347. 10.1038/sj.gt.3301905, 12595892

[bib19] Li, X., Yu, B., Sun, Q., Zhang, Y., Ren, M., Zhang, X., … Qiu, Z. (2018). Generation of a whole-brain atlas for the cholinergic system and mesoscopic projectome analysis of basal forebrain cholinergic neurons. Proceedings of the National Academy of Sciences, 115(2), 415–420. 10.1073/pnas.1703601115, 29259118 PMC5777024

[bib20] Llano, D. A., & Sherman, S. M. (2008). Evidence for nonreciprocal organization of the mouse auditory thalamocortical-corticothalamic projection systems. Journal of Comparative Neurology, 507(2), 1209–1227. 10.1002/cne.21602, 18181153

[bib21] Lotfollahi, M., Naghipourfar, M., Theis, F. J., & Alexander Wolf, F. (2020). Conditional out-of-sample generation for unpaired data using transfer VAE. Bioinformatics, 36, i610–i617. 10.1093/bioinformatics/btaa800, 33381839

[bib22] Mohammadi, S., Ravindra, V., Gleich, D. F., & Grama, A. (2018). A geometric approach to characterize the functional identity of single cells. Nature Communications, 9(1), 1516. 10.1038/s41467-018-03933-2, 29666373 PMC5904143

[bib23] Muzerelle, A., Scotto-Lomassese, S., Bernard, J. F., Soiza-Reilly, M., & Gaspar, P. (2016). Conditional anterograde tracing reveals distinct targeting of individual serotonin cell groups (B5–B9) to the forebrain and brainstem. Brain Structure and Function, 221(1), 535–561. 10.1007/s00429-014-0924-4, 25403254 PMC4750555

[bib24] Oh, S. W., Harris, J. A., Ng, L., Winslow, B., Cain, N., Mihalas, S., … Zeng, H. (2014). A mesoscale connectome of the mouse brain. Nature, 508(7495), 207–214. 10.1038/nature13186, 24695228 PMC5102064

[bib25] Perry, P. O. (2009). Cross-validation for unsupervised learning. arXiv:0909.3052. 10.48550/arXiv.0909.3052

[bib27] Ren, J., Friedmann, D., Xiong, J., Liu, C. D., Ferguson, B. R., Weerakkody, T., … Luo, L. (2018). Anatomically defined and functionally distinct dorsal raphe serotonin sub-systems. Cell, 175(2), 472–487. 10.1016/j.cell.2018.07.043, 30146164 PMC6173627

[bib28] Ren, J., Isakova, A., Friedmann, D., Zeng, J., Grutzner, S. M., Pun, A., … Luo, L. (2019). Single-cell transcriptomes and whole-brain projections of serotonin neurons in the mouse dorsal and median raphe nuclei. eLife, 8, e49424. 10.7554/eLife.49424, 31647409 PMC6812963

[bib29] Salha, R. B., & El Shekh Ahmed, H. I. (2015). Reweighted Nadaraya-Watson estimator of the regression mean. International Journal of Statistics and Probability, 4(1). 10.5539/ijsp.v4n1p138

[bib30] Saul, L. K., & Roweis, S. T. (2003). Think globally, fit locally: Unsupervised learning of low dimensional manifolds. Journal of Machine Learning Research, 4, 119–155.

[bib31] Saunders, A., Johnson, C. A., & Sabatini, B. L. (2012). Novel recombinant adeno-associated viruses for Cre activated and inactivated transgene expression in neurons. Frontiers in Neural Circuits, 6, 47. 10.3389/fncir.2012.00047, 22866029 PMC3406316

[bib32] Wang, Q., Ding, S.-L., Li, Y., Royall, J., Feng, D., Lesnar, P., … Ng, L. (2020). The Allen Mouse Brain Common Coordinate Framework: A 3D reference atlas. Cell, 181(4), 936–953. 10.1016/j.cell.2020.04.007, 32386544 PMC8152789

[bib33] Watson, C., Paxinos, G., & Puelles, L. (2012). The mouse nervous system. Academic Press. 10.1016/B978-0-12-369497-3.10021-4

[bib34] Wu, S., Joseph, A., Hammonds, A. S., Celniker, S. E., Yu, B., & Frise, E. (2016). Stability-driven nonnegative matrix factorization to interpret spatial gene expression and build local gene networks. Proceedings of the National Academy of Sciences, 113(16), 4290–4295. 10.1073/pnas.1521171113, 27071099 PMC4843452

[bib35] Zaborszky, L., Csordas, A., Mosca, K., Kim, J., Gielow, M. R., Vadasz, C., & Nadasdy, Z. (2015). Neurons in the basal forebrain project to the cortex in a complex topographic organization that reflects corticocortical connectivity patterns: An experimental study based on retrograde tracing and 3D reconstruction. Cerebral Cortex, 25(1), 118–137. 10.1093/cercor/bht210, 23964066 PMC4259277

