# Supplementary material for: Modeling the cell-type-specific mesoscale murine connectome with anterograde tracing experiments
Source: Netw Neurosci. 2023 Dec 22;7(4):1497–512. doi: 10.1162/netn_a_00337 (PMC10745083; doi:10.1162/netn_a_00337)
Supplement: Supplementary file 1 [file netn-7-4-1497-s001.pdf]

This supplement is divided into information about our dataset, supplemental methods, and supplemental results. However, certain topics are revisited between sections. Thus, if a reader is interested in, say, non-negative matrix factorization, they may find relevant information in both methods and results.

## 5 SUPPLEMENTAL INFORMATION

Our supplementary information consists of abundances of leaf/Cre-line combinations, information about distances between structures, and the size of our restricted evaluation dataset.

### *Cre/structure combinations in $\mathscr{D}$*

This section describes the abundances of structure and Cre-line combinations in our dataset. That is, it indicates how many experiments in our dataset with a particular Cre-line have an injection centroid in a particular structure. Users of the connectivity matrices who are interested in a particular Cre-line or structure can see the quantity and type of data used to compute and evaluate that connectivity.

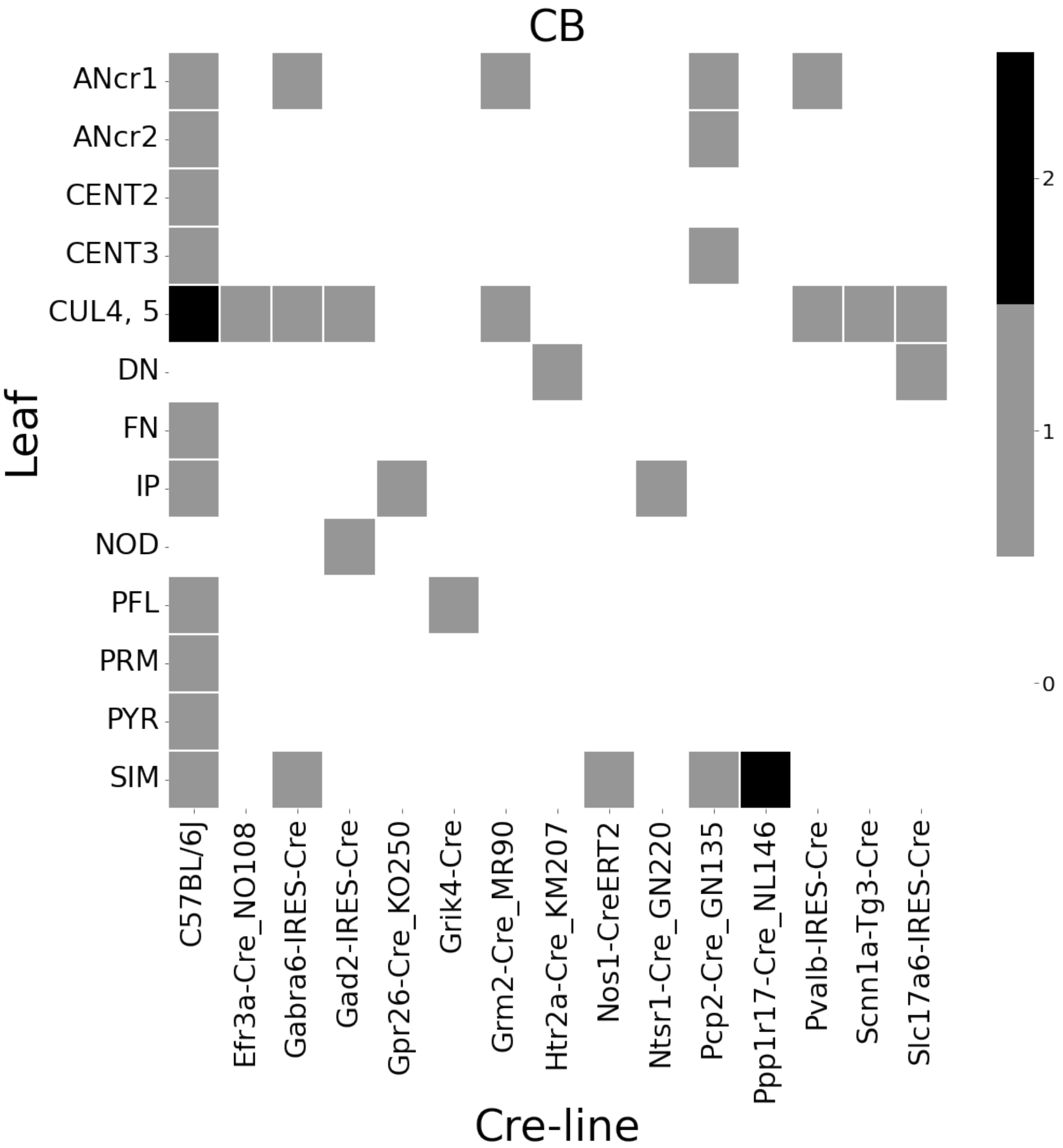


Figure 5: Frequencies of Cre-line and leaf-centroid combinations in our dataset.

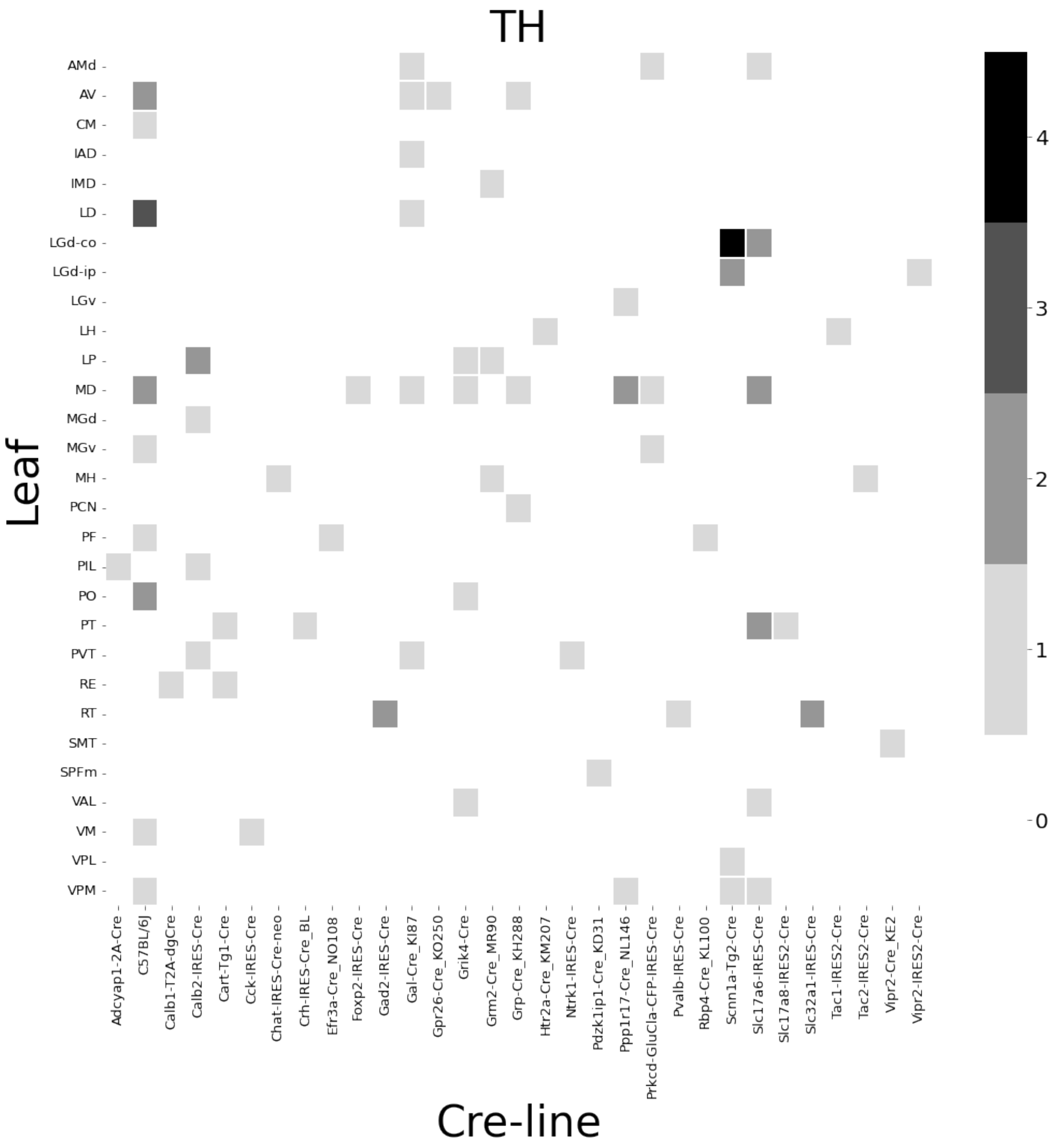


Figure 6: Frequencies of Cre-line and leaf-centroid combinations in our dataset.

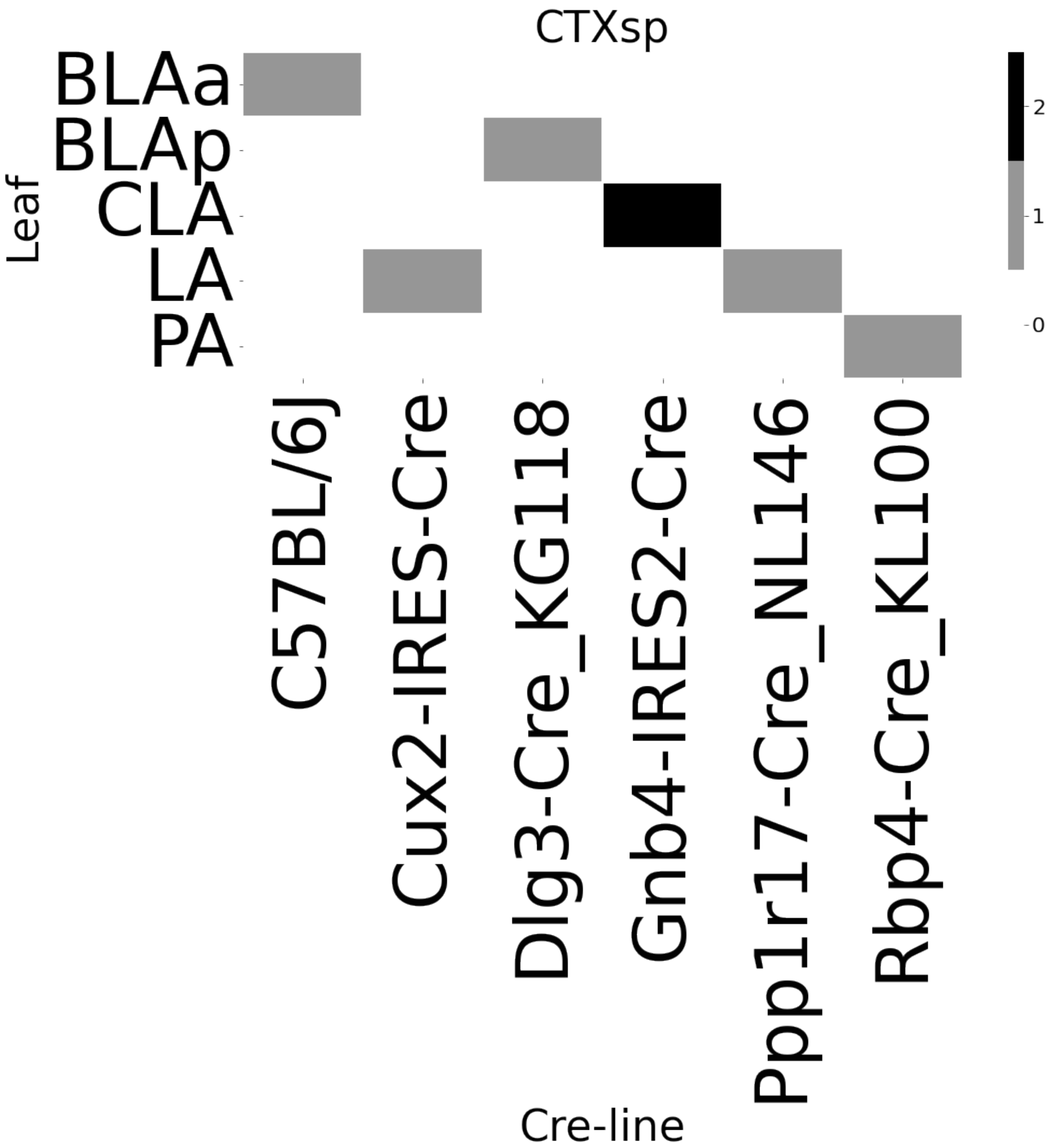


Figure 7: Frequencies of Cre-line and leaf-centroid combinations in our dataset.

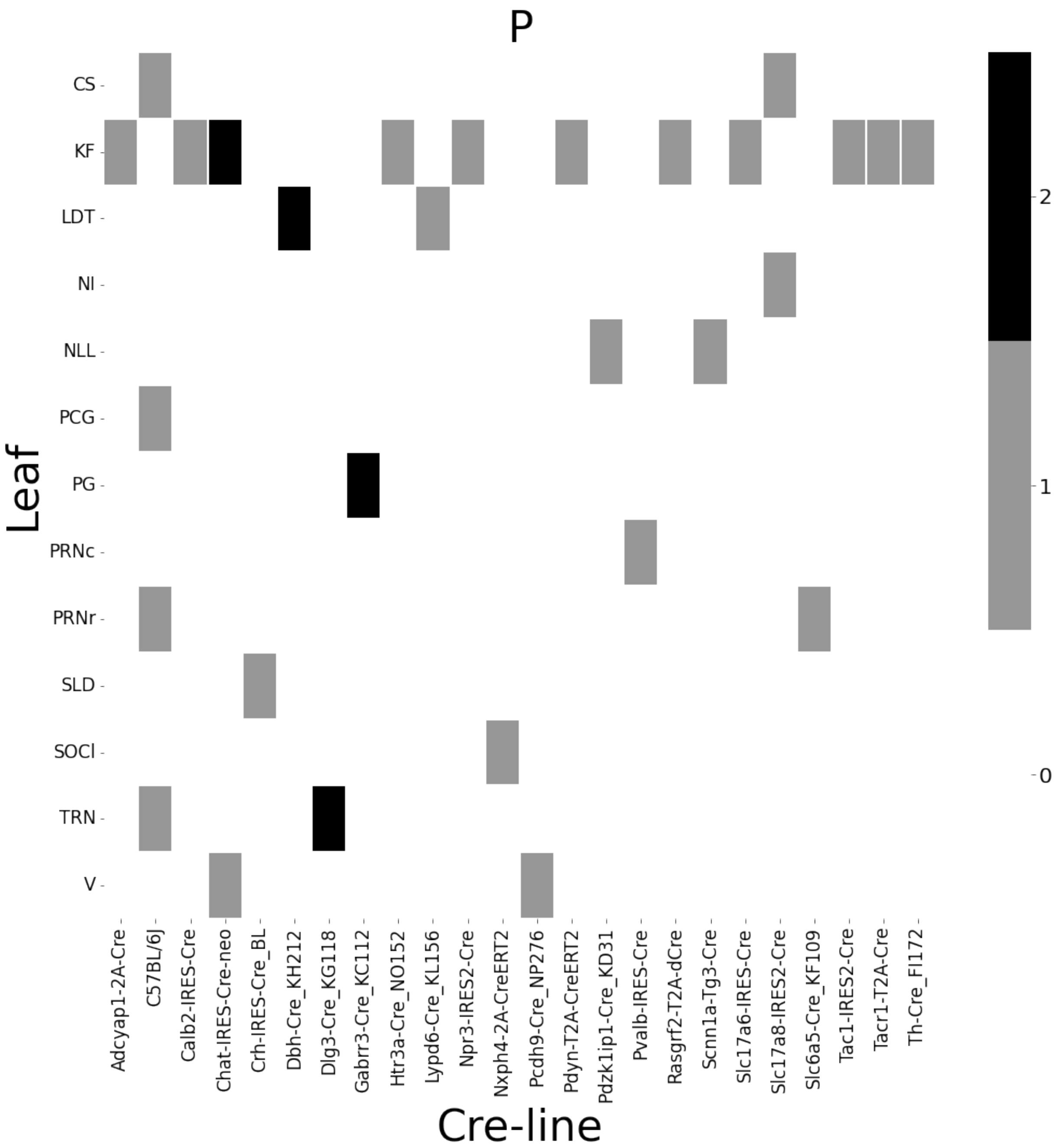


Figure 8: Frequencies of Cre-line and leaf-centroid combinations in our dataset.

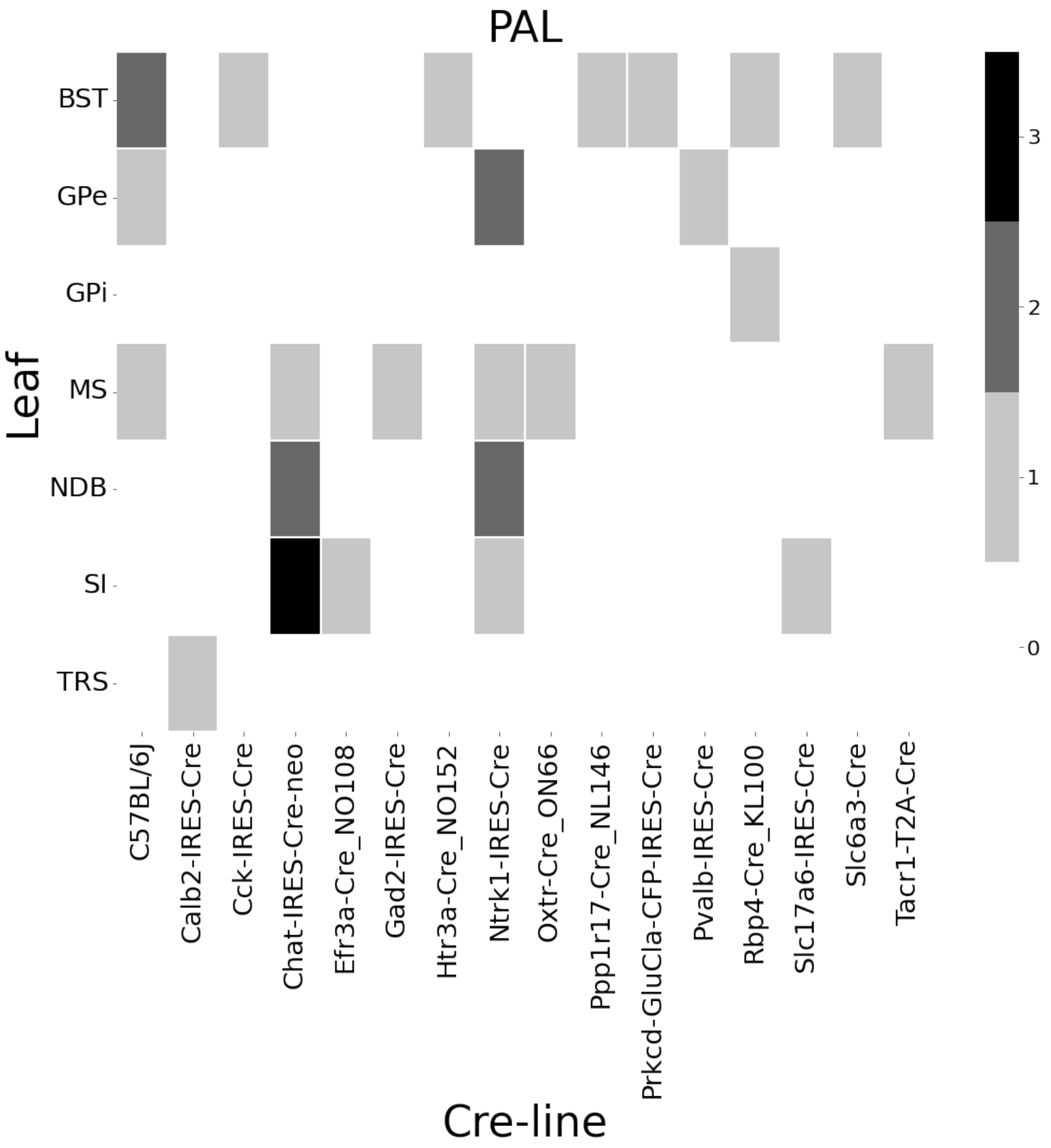


Figure 9: Frequencies of Cre-line and leaf-centroid combinations in our dataset.

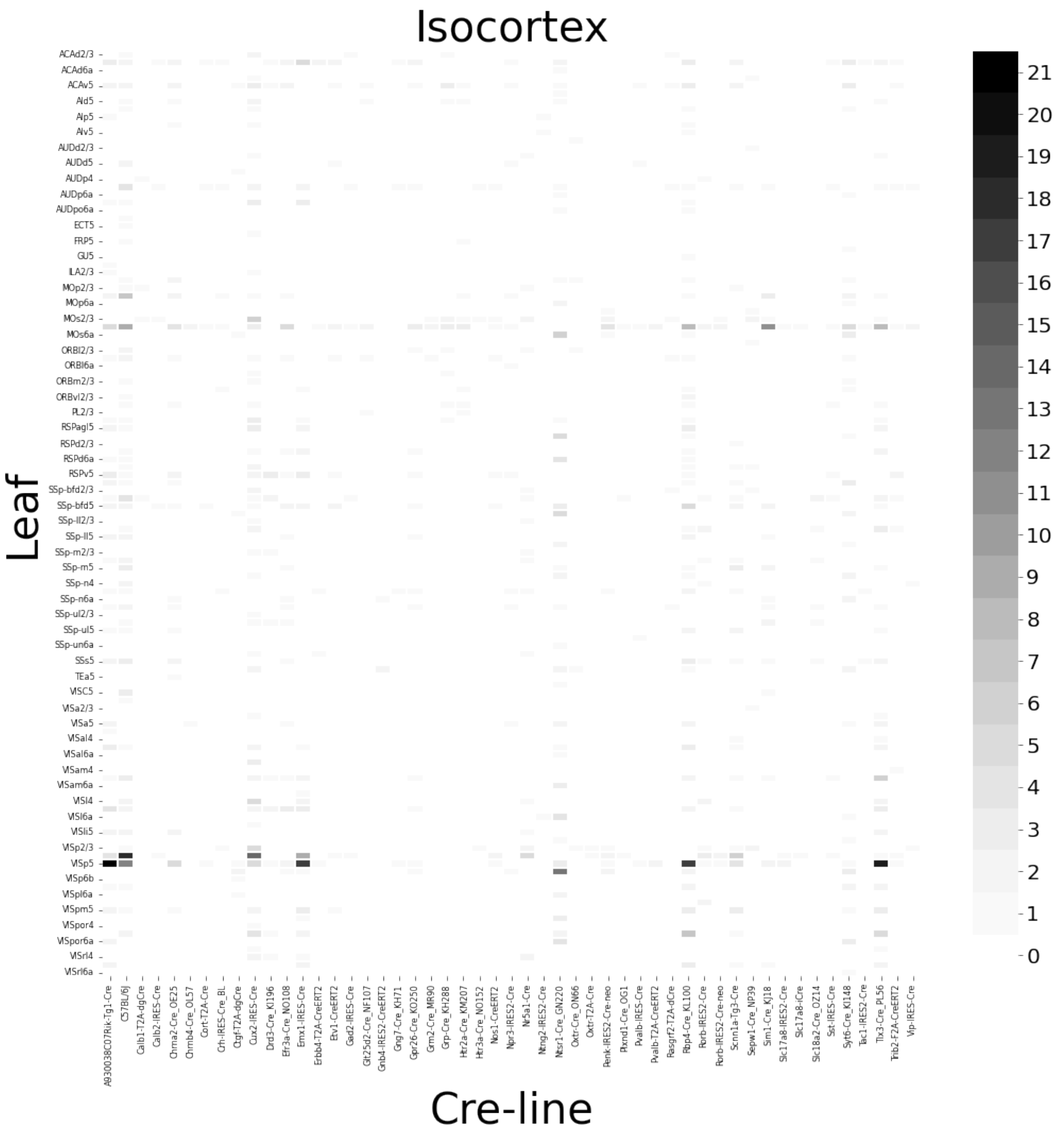


Figure 10: Frequencies of Cre-line and leaf-centroid combinations in our dataset.

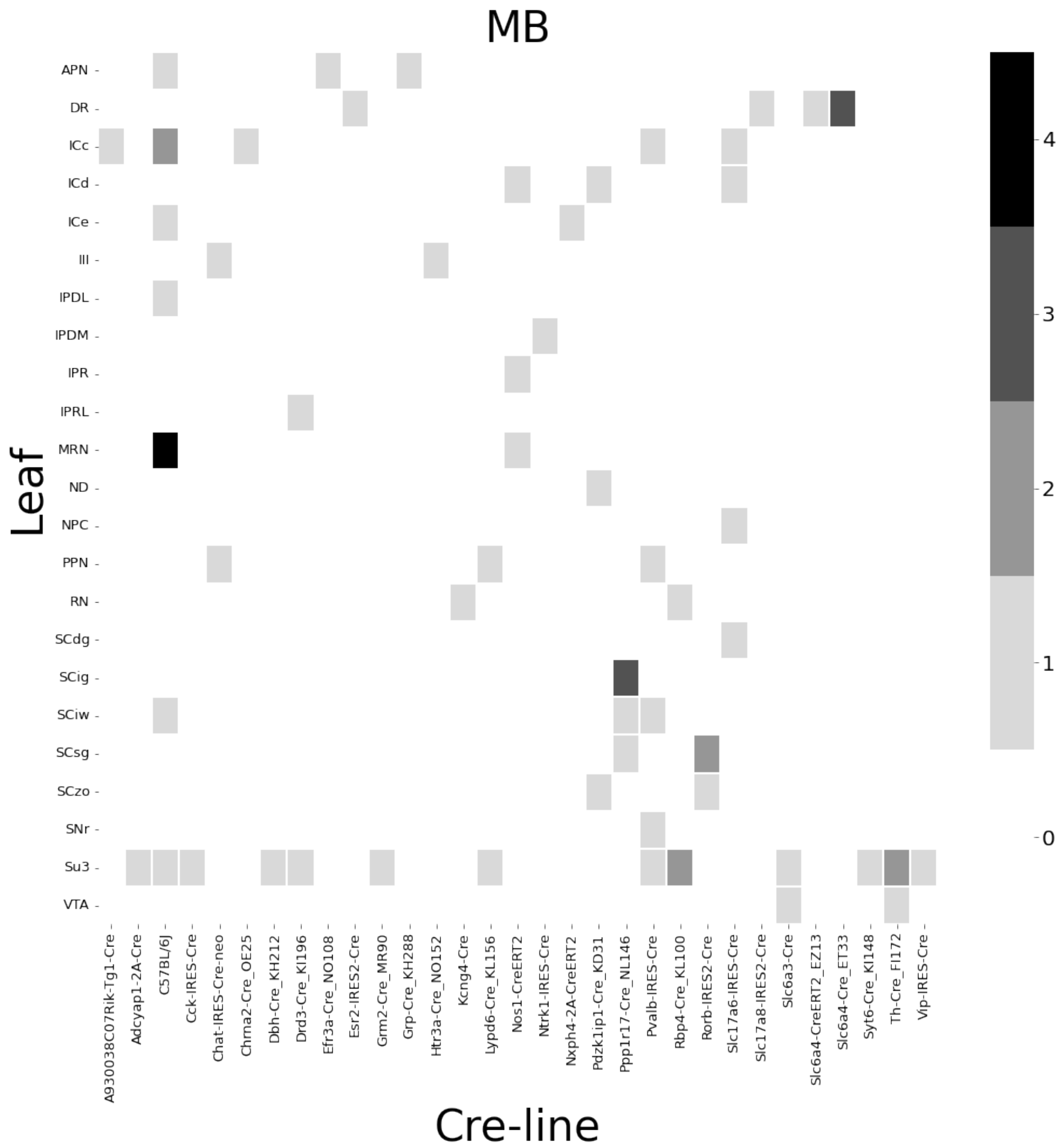


Figure 11: Frequencies of Cre-line and leaf-centroid combinations in our dataset.

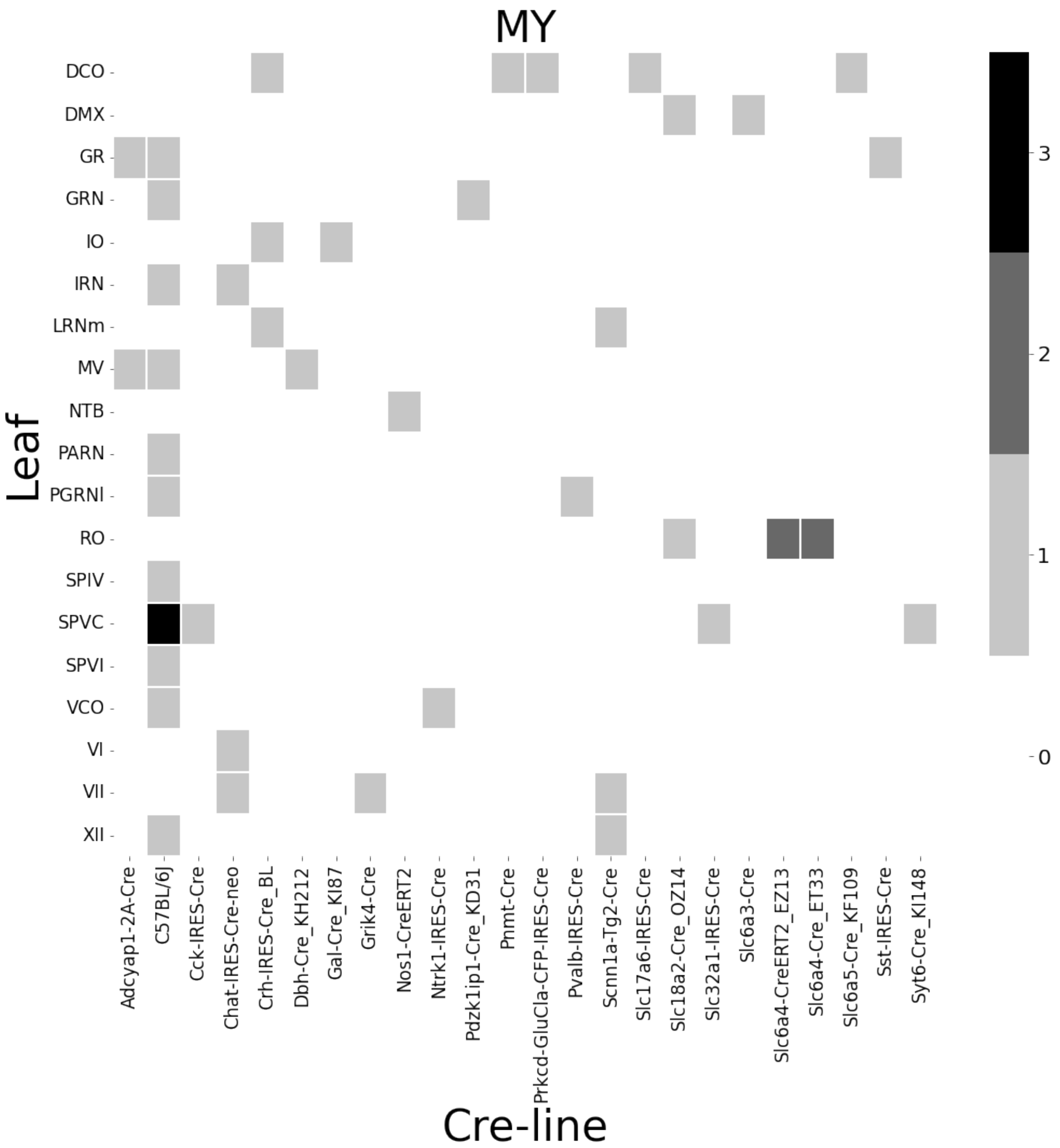


Figure 12: Frequencies of Cre-line and leaf-centroid combinations in our dataset.

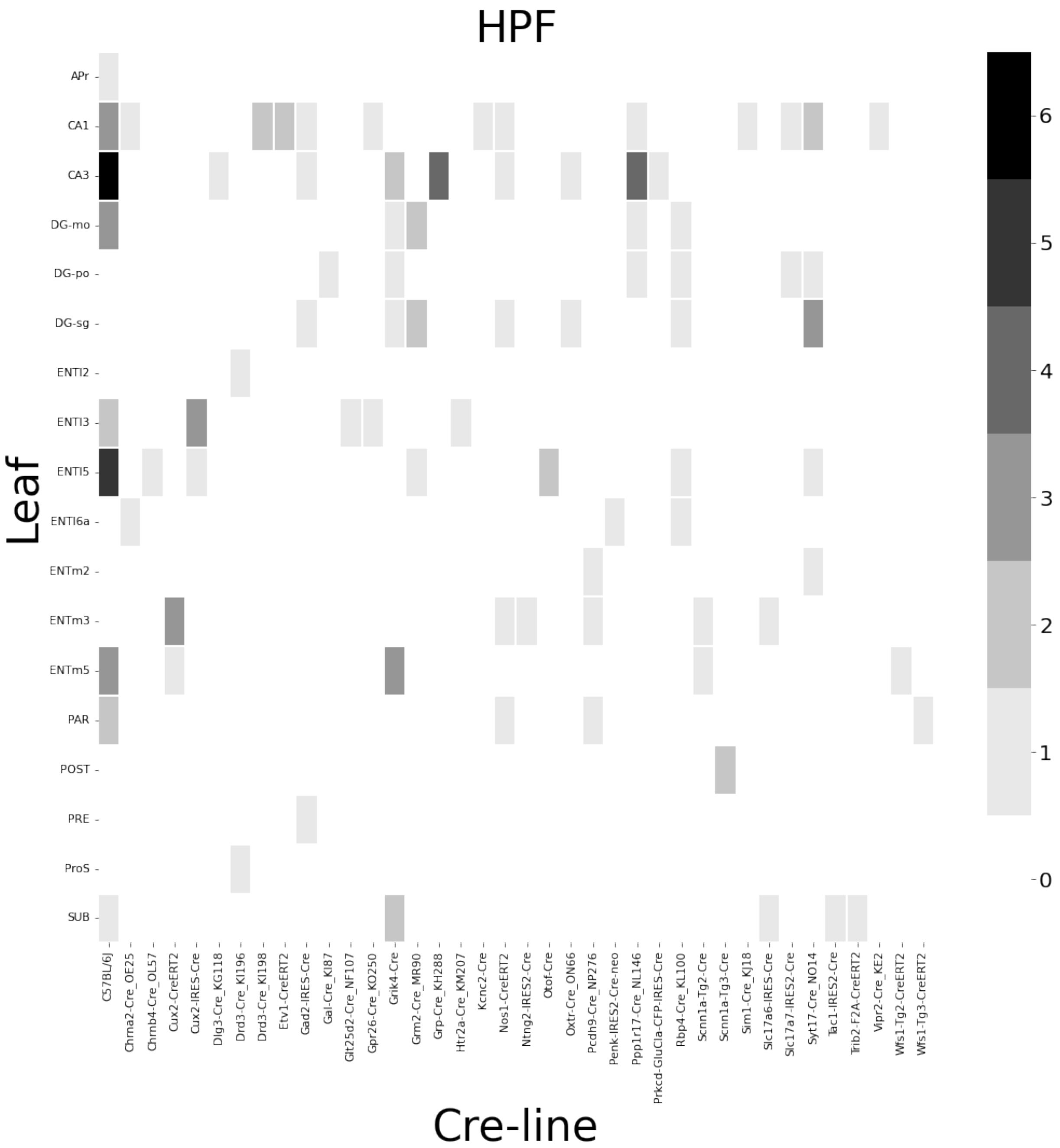


Figure 13: Frequencies of Cre-line and leaf-centroid combinations in our dataset.

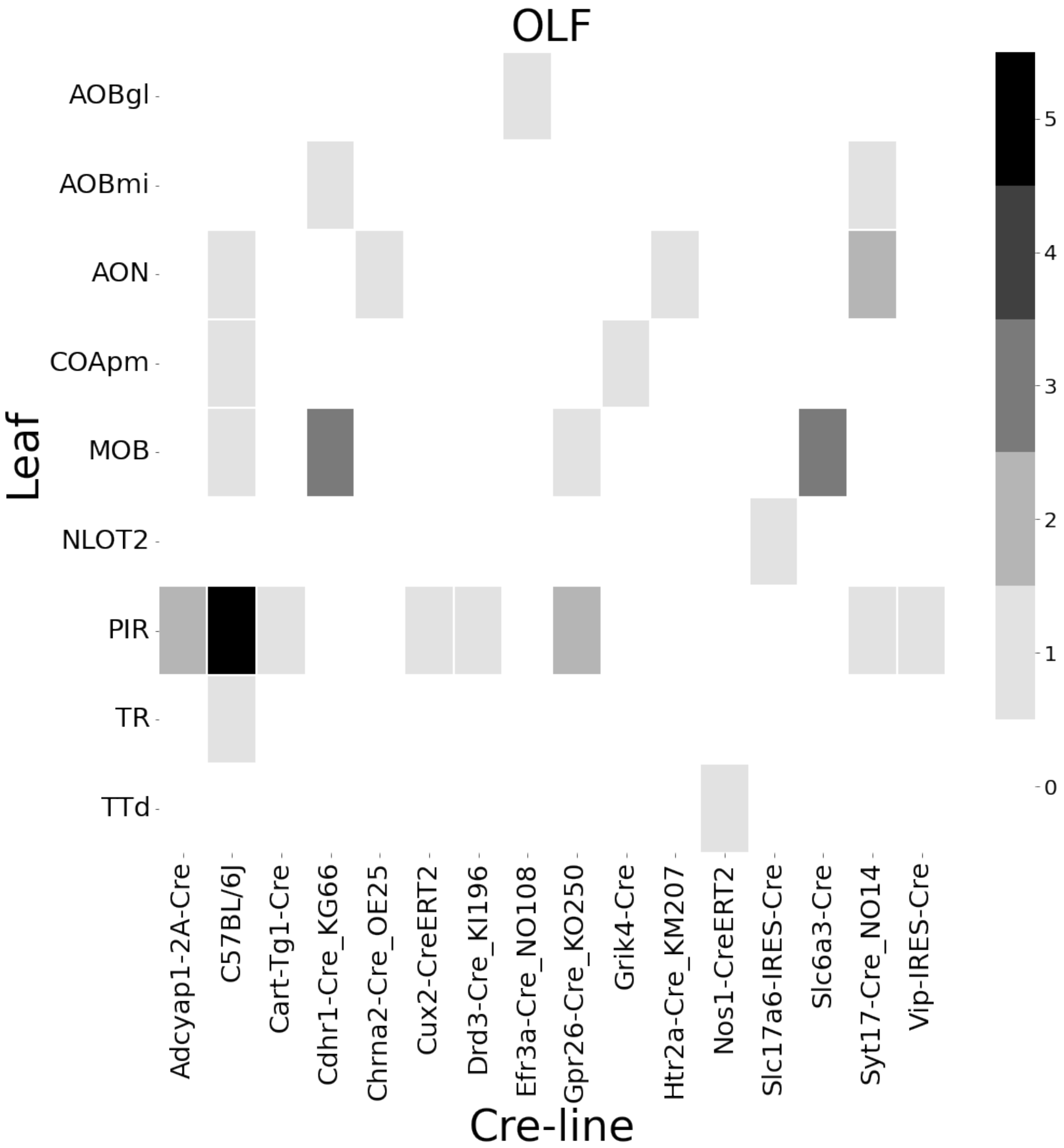


Figure 14: Frequencies of Cre-line and leaf-centroid combinations in our dataset.

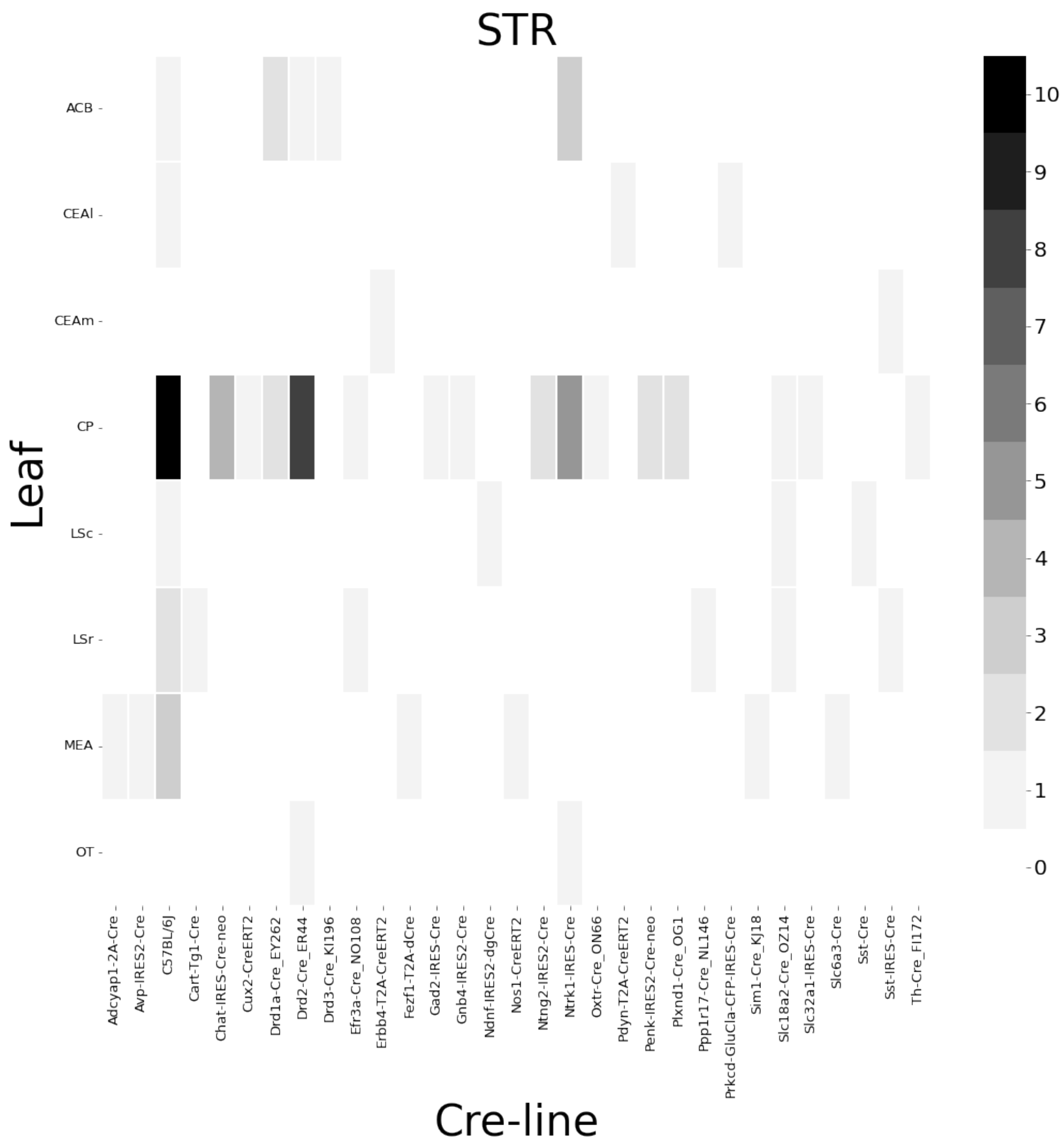


Figure 15: Frequencies of Cre-line and leaf-centroid combinations in our dataset.

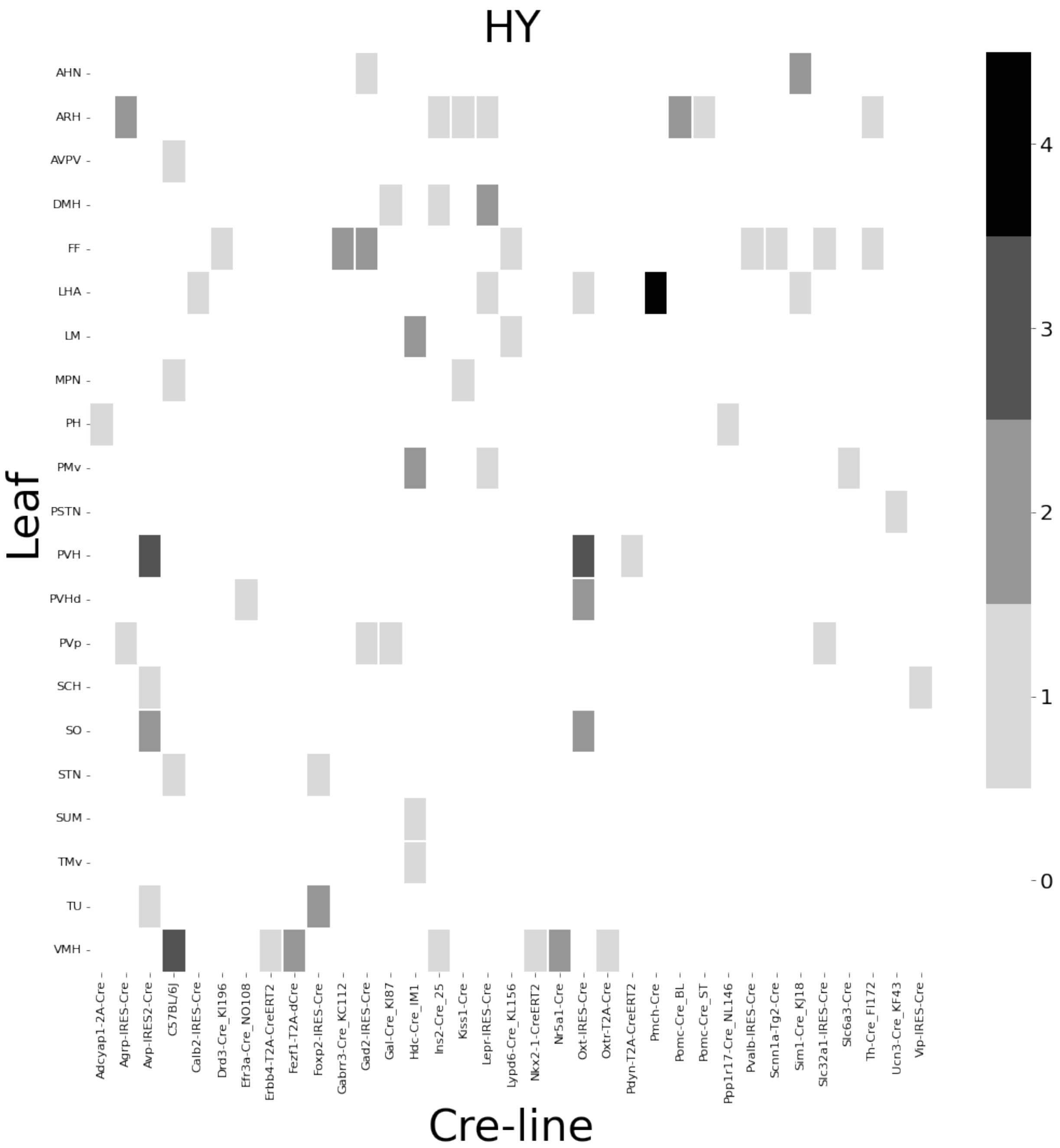


Figure 16: Frequencies of Cre-line and leaf-centroid combinations in our dataset.

### *Distances between structures*

The distance between structures has a strong effect on the connectivity (Knox et al., 2019). For reference, we show these distances here. Short range distances are not used in our matrix factorization approach. This masking is methodologically novel.

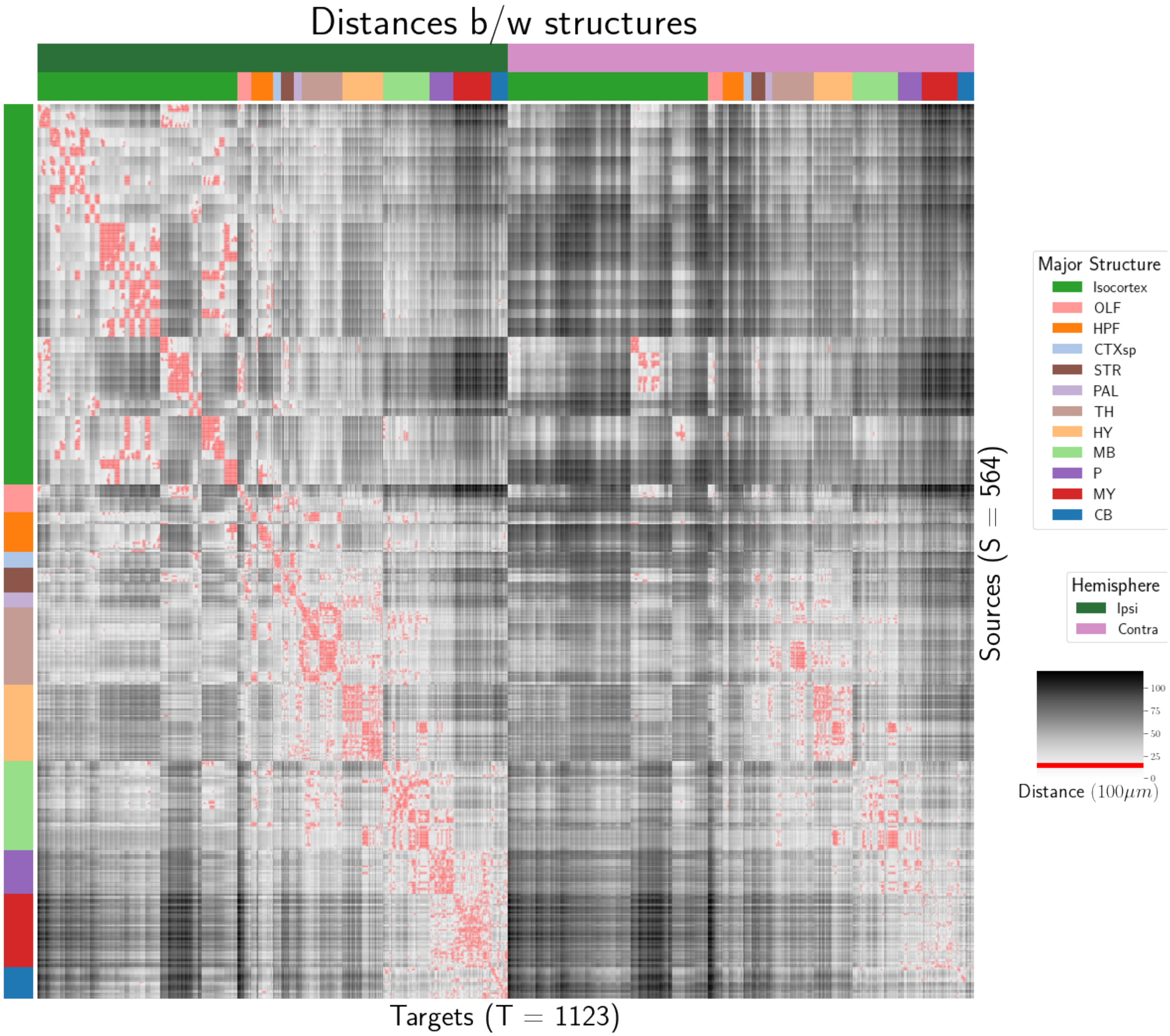


Figure 17: Distance between structures. Short-range connections are masked in red.

### *Model evaluation*

We give the sizes of our evaluation sets in leave-one-out cross-validation and additional losses using the injection-based normalization scheme from Knox et al. (2019).

Number of experiments in evaluation sets In order to compare between methods, we restrict to the smallest set of evaluation indices. That is, the set of experiments used to validate our models combinations that are those whose combination of Cre-line and injection centroid leaf are present at least twice, since one experiment at least must be held out. This means that our evaluation set is smaller in size than our overall list of experiments.

| | Total | Evaluation set | Injection-thresholded evaluation |
|---|---|---|---|
| Isocortex | 1128 | 732 | 709 |
| OLF | 35 | 17 | 17 |
| HPF | 122 | 62 | 59 |
| CTXsp | 7 | 2 | 2 |
| STR | 78 | 45 | 44 |
| PAL | 30 | 11 | 11 |
| TH | 83 | 29 | 29 |
| HY | 85 | 41 | 38 |
| MB | 68 | 18 | 18 |
| P | 33 | 8 | 8 |
| MY | 46 | 7 | 7 |
| CB | 36 | 4 | 4 |

Table 3: Number of experiments available to evaluate models in leave-one-out cross validation. The size of the evaluation set is lower than the total number of experiments since models that rely on a finer granularity of modeling have less data available to validate with, and we restrict all models to the smallest evaluation set necessitated by any of the modes. In this case, the Expected Loss and Cre-NW models require at least two experiments to be present with a combination of injection centroid structure and Cre-line for leave-one-out cross-validation. We also include results with a slightly smaller evaluation set that removes experiments without a sufficiently strong injection signal, as in Knox et al. (2019)

Injection-normalized losses To compare with the injection-normalization procedure from Knox et al. (2019), we also remove experiments with small injection, and here give results for this slightly reduced set using injection-normalization. That is, instead of dividing the projection signal of each experiment by its $l1$ norm (as we have used throughout the study), we divide by the $l1$ norm of the corresponding injection signal. We find that setting a summed injection-signal of threshold of 1 is sufficient for evading pathological edge cases in this normalization, while still retaining a large evaluation set.

| $\widehat{f}$ | Mean | NW | | | | | EL |
|---|---|---|---|---|---|---|---|
| $\mathscr{D}$ | $I_c \cap I_L$ | $I_c \cap I_M$ | $I_c \cap I_L$ | $I_L$ | $I_{wt} \cap I_M$ | $I_M$ | $I_L$ |
| Isocortex | 0.413 | 0.453 | 0.408 | 0.538 | 0.528 | 0.528 | **0.396** |
| OLF | 0.499 | 0.504 | 0.494 | 0.441 | 0.543 | 0.543 | **0.437** |
| HPF | 0.336 | 0.483 | 0.332 | 0.444 | 0.501 | 0.501 | **0.321** |
| CTXsp | 0.497 | 0.497 | 0.497 | 0.497 | 0.497 | 0.497 | 0.497 |
| STR | 0.359 | 0.386 | 0.359 | 0.364 | 0.433 | 0.433 | **0.322** |
| PAL | 0.519 | 0.497 | 0.519 | 0.436 | 0.459 | 0.459 | **0.434** |
| TH | 0.769 | 0.767 | 0.769 | **0.514** | 0.539 | 0.539 | 0.556 |
| HY | 0.414 | 0.439 | 0.414 | 0.441 | 0.452 | 0.452 | **0.399** |
| MB | 0.459 | 0.396 | 0.397 | 0.358 | **0.324** | **0.324** | 0.403 |
| P | **0.562** | **0.562** | **0.562** | 0.758 | 0.764 | 0.764 | **0.562** |
| MY | 0.699 | 0.552 | 0.621 | **0.439** | 0.578 | 0.578 | **0.439** |
| CB | 0.849 | 0.689 | 0.849 | 0.500 | 0.615 | 0.615 | **0.495** |

Table 4: Losses from leave-one-out cross-validation of candidate for injection-normalized regionalized connectivity on injection-thresholded evaluation set. **Bold** numbers are best for their major structure.

420 Projection-normalized losses on thresholded set We also give results for the
421 projection-normalization procedure from the main text on this reduced subset.

| $\widehat{f}$ | Mean | NW | | | | | EL |
|---|---|---|---|---|---|---|---|
| $\mathscr{D}$ | $I_c \cap I_L$ | $I_c \cap I_M$ | $I_c \cap I_L$ | $I_L$ | $I_{wt} \cap I_M$ | $I_M$ | $I_L$ |
| Isocortex | 0.229 | 0.248 | 0.224 | 0.274 | 0.269 | 0.269 | **0.217** |
| OLF | 0.193 | 0.233 | 0.191 | **0.135** | 0.179 | 0.179 | 0.138 |
| HPF | 0.178 | 0.342 | **0.172** | 0.212 | 0.235 | 0.235 | **0.172** |
| CTXsp | **0.621** | **0.621** | **0.621** | **0.621** | **0.621** | **0.621** | **0.621** |
| STR | 0.128 | **0.117** | 0.124 | 0.171 | 0.234 | 0.234 | 0.125 |
| PAL | 0.203 | 0.205 | 0.203 | 0.295 | 0.291 | 0.291 | **0.188** |
| TH | 0.673 | 0.664 | 0.673 | **0.358** | 0.379 | 0.379 | 0.417 |
| HY | 0.358 | 0.378 | 0.351 | 0.331 | 0.312 | **0.312** | 0.314 |
| MB | 0.168 | 0.191 | **0.160** | 0.199 | 0.202 | 0.202 | **0.160** |
| P | 0.292 | 0.292 | 0.292 | 0.299 | 0.299 | 0.299 | **0.287** |
| MY | 0.268 | 0.347 | 0.268 | **0.167** | 0.189 | 0.189 | 0.196 |
| CB | 0.062 | 0.062 | 0.062 | 0.068 | 0.108 | 0.108 | **0.061** |

Table 5: Losses from leave-one-out cross-validation of candidate for normalized regionalized connectivity on injection-thresholded evaluation set. **Bold** numbers are best for their major structure.

# 6 SUPPLEMENTAL METHODS

This section consists of additional information on preprocessing of the neural connectivity data, estimation of connectivity, and matrix factorization.

## *Data preprocessing*

Several data prepreprocessing steps take place prior to evaluations of the connectivity matrices. These steps are described in Algorithm PREPROCESS. The arguments of this normalization process - injection signals $x(i)$, projection signals $y(i)$, injection fraction $F(i)$, and data quality mask $q(i)$ - were downloaded using the Allen SDK, a programatic interface to the brain connectivity data. The injections and projection signals $\mathcal{B} \to [0,1]$ were segmented manually in histological analysis. The projection signal gives the proportion of pixels within the voxel displaying fluorescence, and the injection signal gives the proportion of pixels within the histologically-selected injection subset displaying fluorescence. The injection fraction $F(i) : \mathcal{B} \to [0,1]$ gives the proportion of pixels within each voxel in the injection subset. Finally, the data quality mask $q(i) : \mathcal{B} \to \{0,1\}$ gives the voxels that have valid data.

Our preprocessing makes use of the above ingredients, as well as several other essential steps. First, we compute the weighted injection centroid

$$c(i) \quad = \sum_{l \in \mathcal{B}} x(i)|_l$$

where $x(i)|_l$ is the injection density at location $l \in \mathbb{R}^3$. Given a regionalization $\mathcal{R}$ from the Allen SDK, we can also access regionalization map $R : \mathcal{B} \to \mathcal{R}$. This induces a functional of connectivities from the space of maps $\{\mathcal{X} = x : \mathcal{B} \to [0,1]\}$

$$\begin{aligned} 1_{\mathcal{R}} : \mathcal{X} &\to \mathcal{R} \times \mathbb{R}_{\geq 0} \\ x &\mapsto \sum_{l \in r} x(l) \text{ for } r \in \mathcal{R}. \end{aligned}$$

We also can restrict a signal to a individual structure as

$$\begin{aligned} 1|_s : \mathscr{X} &\to \mathscr{X} \\ x(l) &= \begin{cases} x(l) \text{ if } l \in S \\ 0 \text{ otherwise .} \end{cases} \end{aligned}$$

Finally, given a vector or array $a \in \mathbb{R}^T$, we have the $l1$ normalization map

$$n : a \mapsto \frac{a}{\sum_{j=1}^{T} a_j}.$$

Denote $m$ as the major structure containing an experiment, and define $\odot$ for maps $\mathscr{B} \to [0,1]$ by e.g. $(y(i) \odot q(i))|_l := (y(i)|_l)(q(i)|_l)$. We then can write the preprocessing algorithm.

**PREPROCESS 1 Input** Injection $x$, Projection $y$, Injection centroid $c \in \mathbb{R}^3$, Injection fraction $F$, data quality mask $q$

Injection fraction $x_F \leftarrow x \odot F$

Data-quality censor $y_q \leftarrow y \odot q, x_q \leftarrow x_F \odot q$

Restrict injection $x_m = 1|_m x_q$.

Compute centroid $c$ from $x_m$

Regionalize $\tilde{y}_{\mathscr{T}} \leftarrow 1_{\mathscr{T}}(y_q)$

Normalize $y_{\mathscr{T}} \leftarrow n(\tilde{y}_{\mathscr{T}})$

**Output** $\tilde{y}_{\mathscr{T}}, c$

### *Estimators*

As mentioned previously, we can consider our estimators as modeling a connectivity vector $f_{\mathcal{T}}(v,s) \in \mathbb{R}^T_{\geq 0}$. Thus, for the remainder of this section, we will discuss only $f(v,s)$. We review the Nadaraya-Watson estimator from Knox et al. (2019), and describe its conversion into our cell-class specific Expected Loss estimator.

*Centroid-based Nadaraya-Watson* In the Nadaraya-Watson approach of Knox et al. (2019), the injection is considered only through its centroid $c(i)$, and the projection is considered regionalized. That is,

$$f_*(i) = \{c(i), y_{\mathcal{T}}(i)\}.$$

Since the injection is considered only by its centroid, this model only generates predictions for particular locations $l$, and the prediction for a structure $s$ is given by integrating over locations within the structure

$$f^*(\hat{f}(f_*(\mathcal{D})))(v,s) = \sum_{l \in s} \hat{f}(f_*(\mathcal{D}(I)))(v,l).$$

Here, $I$ is the training data, and $\hat{f}$ is the Nadaraya-Watson estimator

$$\hat{f}_{NW}(c(I), y_{\mathcal{T}}(I))(l) := \sum_{i \in I} \frac{\omega_{il}}{\sum_{i \in I} \omega_{il}} y_{\mathcal{T}}(i)$$

where $\omega_{il} := \exp(-\gamma d(l, c(i))^2)$ and $d$ is the Euclidean distance between centroid $c(i)$ and voxel with position $l$.

Several facets of the estimator are visible here. A smaller $\gamma$ corresponds to a greater amount of smoothing, and the index set $I \subseteq \{1:n\}$ generally depends on $s$ and $v$. Varying $\gamma$ bridges between 1-nearest neighbor prediction and averaging of all experiments in $I$. In Knox et al. (2019), $I$ consisted of experiments sharing the same brain division, i.e. $I = I_m$, while restricting the index set to only include experiments with the same cell class gives the class-specific Cre-NW model. Despite this restriction, we fit $\gamma$ by leave-one-out cross-validation for each $m$ rather than a smaller subset like $s$ or $v$. That is,

$$\widehat{\gamma}_m = \arg\min_{\gamma \in \mathbb{R}_{\geq 0}} \frac{1}{|\{s,v\}|} \sum_{s,v \in \{m, \mathcal{V}\}} \frac{1}{|I_s \cap I_v|} \sum_{i \in (I_s \cap I_v)} \ell(y_{\mathcal{T}}(i)), \hat{f}_{\mathcal{T}}(f_*(\mathcal{D}(v,s) \setminus i)). \quad (2)$$

*The Expected-Loss estimator* Besides location of the injection centroid, cell class also influences projection. Thus, we introduce method for estimating the effect of Cre-distance, which we define as the distance between the projections of the mean experiment of one (Cre,leaf) pair with another. Equivalently, relatively small Cre-distance defines what we call similar cell classes. This method assigns a predictive weight to each pair of training points that depends both on their centroid-distance and Cre-distance. This weight is determined by the expected prediction error of each of the two feature types

We define Cre-line behavior as the average regionalized projection of a Cre-line in a given structure (i.e. leaf). The vectorization of categorical information is known as **target encoding**

$$\bar{y}_{\mathcal{T},s,v} := \frac{1}{|I_s \cap I_v|} \sum_{i \in (I_s \cap I_v)} y_{\mathcal{T}}(i)$$

We then define a **Cre-distance** in a leaf to be the distance between the target-encoded projections of two Cre-lines. The relative predictive accuracy of Cre-distance and centroid distance is determined by fitting a surface of projection distance as a function of Cre-distance and centroid distance. For this reason, we call this the Expected Loss Estimator. When we use shape-constrained B-splines to estimate this weight, the weights then may be said to be used in a Nadaraya-Watson estimator. The resulting weights are then utilized in a Nadaraya-Watson estimator in a final prediction step.

In mathematical terms, our full feature set consists of the centroid coordinates and the target-encoded means of the combinations of virus type and injection-centroid structure. That is,

$$f_*(\mathcal{D}_i) = \{c(i), \{\bar{y}_{\mathcal{T},s,v} \forall v\}, y_{\mathcal{T}}(i)\}.$$

$f^*$ is defined as in (2). The expected loss estimator is then

$$\hat{f}_{EL}(c(I), y_{\mathcal{T}}(I))(l,v) := \sum_{i \in I} \frac{\nu_{ilv}}{\sum_{i \in I} \nu_{ilv}} y_{\mathcal{T}}(i)$$

where

$$\nu_{ilv} := \exp(-\gamma g(d(l,c(i))^2, d(\bar{y}_{\mathcal{T},s,v}, \bar{y}_{\mathcal{T},s,v(i)})^2))$$

and $s$ is the structure containing $l$.

The key step therefore is finding a suitable function $g$ with which to weight the positional and (Cre,leaf) information. Note that $g$ must be a concave, non-decreasing function of its arguments with with $g(0,0) = 0$. Then, $g$ defines a metric on the product of the metric spaces defined by experiment centroid and target-encoded cre-line, and $\hat{f}_{EL}$ is a Nadaraya-Watson estimator. A derivation of this fact is given later in this section.

We therefore use a linear generalized additive model of shape-constrained B-splines to estimate $g$ (Eilers & Marx, 1996). This is a method for generating a predictive model $g$ that minimizes the loss of

$$\sum_{i,i' \in S} \| \| y_{\mathcal{T}}(i) - y_{\mathcal{T}}(i') \|_2 - \sum_{q=1}^{Q} \rho_q B_q(\| c(i') - c(i) \|_2, \| \bar{y}_{\mathcal{T},s,v} - \bar{y}_{\mathcal{T},s,v}(i) \|) \|_2$$

given the constraints on $g$. That is, given all pairs of experiments with injection centroid in the same structure, $g$ gives a prediction of the distance between their projections made using the distance between the average behavior of their Cre-lines given their injection centroid, and the distance between their injection centroids. In particular, $g$ is the empirically best such function within the class of $B$-splines, which Similarly to the Nadaraya-Watson model, we make the decision to fit a $g$ separately for each major brain division, and select $\hat{\gamma}$ as in 2. We set $Q = 10$ and leave validation of this parameter, as well as the precise nature of the polynomial B-spline terms $B_q$ out of the scope of this paper. Empirically this leads to a smooth surface using the pyGAM Python package (Servén & Brummitt, 2018).

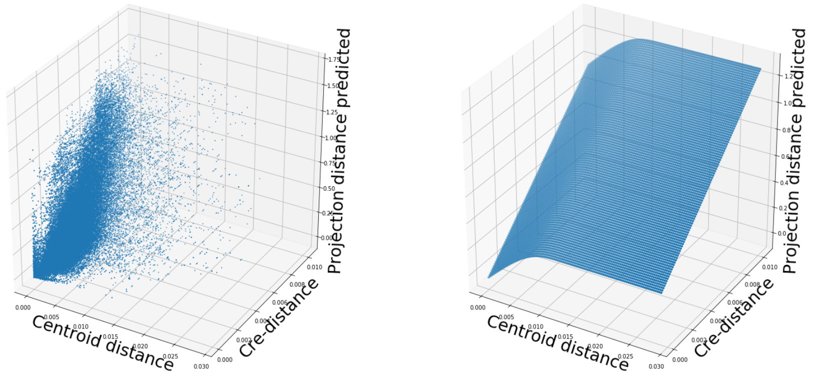


(a) (b)

Figure 18: Fitting $g$. 18a Distribution of projection errors against centroid distance and cre-distance in Isocortex. 18b estimated $\hat{g}$ using B-splines. Projection distance is $\|y_{\mathcal{T}}(i) - y_{\mathcal{T}}(i')\|_2$, Cre-distance is $\|\bar{y}_{\mathcal{T},s,v} - \bar{y}_{\mathcal{T},s,v}(i)\|)\|_2$, and centroid-distance is $\|c(i') - c(i)\|_2$.

Justification of shape constraint The shape-constrained expected-loss estimator introduced in this paper is, to our knowledge, novel. It should be considered an alternative method to the classic weighted kernel method (Cai, 2001; Salha & El Shekh Ahmed, 2015). While we do not attempt a detailed theoretical study of this estimator, we do establish the need for the shape constraint in our spline estimator. Though this fact is probably well known, we prove a (slightly stronger) version here for completeness.

**Proposition 1.** *Given a collection of metric spaces $X_1, ...X_n$ with metrics $d_1 ... d_n$ (e.g. $d_{centroid}, d_cre$), and a function $f : (X_1 \times X_1) ... \times (X_n \times X_n) = g(d_1(X_1 \times X_1), ...d_n(X_n \times X_n))$, then $f$ is a metric if $g$ is concave, non-decreasing and $g(d) = 0 \iff d = 0$.*

*Proof.* We show $g$ satisfying the above properties implies that $f$ is a metric.

- The first property of a metric is that $f(x, x') = 0 \iff x = x'$. The left implication: $x = x' \implies f(x_1, x'_1, ...x_n, x'_n) = g(0, ...., 0)$, since $d$ are metrics. Then, since $g(0) = 0$, we have that $f(x, x') = 0$. The right implication: $f(x, x') = 0 \implies d = 0 \implies x = x'$ since $d$ are metrics.
- The second property of a metric is that $f(x, x') = f(x', x)$. This follows immediately from the symmetry of the $d_i$, i.e. $f(x, x') = f(x_1, x'_1, ...x_n, x'_n) = g(d_1(x_1, x'_1), ...d_n(x_n, x'_n)) = g(d_1(x'_1, x_1), ...d_n(x'_n, x_n)) = f(x'_1, x_1, ...x'_n, x_n) = f(x', x)$.
- The third property of a metric is the triangle inequality: $f(x, x') \leq f(x, x^*) + f(x^*, x')$. To show this is satisfied for such a $g$, we first note that $f(x, x') = g(d(x, x')) \leq g(d(x, x^*) + d(x^*, x'))$ since g is non-decreasing and by the triangle inequality of $d$. Then, since $g$ is concave, $g(d(x, x^*) + d(x^*, x')) \leq g(d(x, x^*)) + g(d(x^*, x')) = f(x, x^*) + f(x^*, x')$.

□

### *Setting a lower detection threshold*

The lower detection threshold of our approach is a complicated consequence of our experimental and analytical protocols. For example, the Nadaraya-Watson estimator is likely to generate many small false positive connections, since the projection of even a single experiment within the source region to a target will cause a non-zero connectivity in the Nadaraya-Watson weighted average. On the other hand, the complexities of the experimental protocol itself and the image analysis and alignment can also cause spurious signals. Therefore, it is of interest to establish a lower-detection threshold below which we have very little power-to-predict, and set estimated connectivities below this threshold to zero.

We set this threshold with respect to the sum of Type 1 and Type 2 errors

$$\iota = \sum_{i \in \mathcal{E}} 1^T_{y_{\mathcal{T}}(i)=0} 1_{\hat{f}_{\mathcal{T}}(v(i),c(i))>\tau} + 1^T_{y_{\mathcal{T}}(i)>0} 1_{\hat{f}_{\mathcal{T}}(v(i),c(i))<\tau}.$$

We then select the $\tau$ that minimizes $\iota$. Results for this approach are given in Supplemental Section 7.

### *Decomposing the connectivity matrix*

We utilize non-negative matrix factorization (NMF) to analyze the principal signals in our connectivity matrix. Here, we review this approach as applied to decomposition of the distal elements of the estimated connectivity matrix $\widehat{\mathscr{C}}$ to identify $q$ connectivity archetypes. Aside from the NMF program itself, the key elements are selection of the number of archetypes $q$ and stabilization of the tendency of NMF to give random results over different initializations.

*Non-negative matrix factorization* As discussed in Knox et al. (2019), one of the most basic processes underlying the observed connectivity is the tendency of each source region to predominantly project to proximal regions. For example, the heatmap in Supplemental Figure 17 shows that the pattern of intrastructure distances resembles the connectivity matrix in 2. These connections are biologically meaningful, but also unsurprising, and their relative strength biases learned latent coordinate representations away from long-range structures. For this reason, we establish a 1500 $\mu m$ 'distal' threshold within which to exclude connections for our analysis.

Given a matrix $X \in \mathbb{R}_{\geq 0}^{a \times b}$ and a desired latent space dimension $q$, the non-negative matrix factorization is thus

$$\text{NMF}(\mathscr{C}, \lambda, q, 1_M) = \arg \min_{W \in \mathbb{R}_{\geq 0}^{S \times q}, H \in \mathbb{R}_{\geq 0}^{q \times T}} \frac{1}{2} \| 1_M \odot \mathscr{C} - WH \|_2^2 + \lambda (\| H \|_1 + \| W \|_1).$$

The mask $1_M$ specifies this objective for detecting patterns in long-range connections. We note the existence of NMF with alternative norms for certain marginal distributions, but leave utilization of this approach for future work (Brunet et al., 2004).

The mask $1_M \in \{0, 1\}^{S \times T}$ serves two purposes. First, it enables computation of the NMF objective while excluding self and nearby connections. These connections are both strong and linearly independent, and so would unduly influence the $NMF$ reconstruction error over more biologically interesting or cell-type dependent long-range connections. Second, it enables cross-validation based selection of the number of retained components.

*Cross-validating NMF* We review cross-validation for NMF following (Perry, 2009). In summary, a NMF model is first fit on a reduced data set, and an evaluation set is held out. After random masking of the evaluation set, the loss of the learned model is then evaluated on the basis of successful reconstruction of the held-out values. This procedure is performed repeatedly, with replicates of random masks at each tested dimensionality $q$. This determines the point past which additional hidden units provide no additional value for reconstructing the original signal.

The differentiating feature of cross-validation for NMF compared with supervised learning is the randomness of the masking matrix $1_M$. Cross-validation for supervised learning generally leaves out entire observations, but this is insufficient for our situation. This is because, given $W$, our $H$ is the solution of a regularized non-negative least squares optimization problem

$$H := \widehat{e}_W(1_M \odot \mathscr{C}) = \arg\min_{\beta \in \mathbb{R}_{\geq 0}^{q \times T}} \|1_M \odot \mathscr{C} - W\beta\|_2^2 + \|\beta\|_1. \tag{3}$$

The negative effects of an overfit model can therefore be optimized away from on the evaluation set.

We therefore generate uniformly random masks $1_{M(p)} \in \mathbb{R}^{S \times T}$ where

$$1_{M(p)}(s,t) \sim \text{Bernoulli(p)}.$$

NMF is then performed using the mask $1_{M(p)}$ to get $W$. The cross-validation error is then

$$\epsilon_q = \frac{1}{R}\sum_{r=1}^{R}(\|1_{M(p)_r^C} \odot X - W(\widehat{e}_W(1_{M(p)_r^C} \odot X))\|_2^2$$

where $1^C_{M(p)_r}$ is the binary complement of $1_{M(p)_r}$ and $R$ is a number of replicates. Theoretically, the optimum number of components is then

$$\widehat{q} = \arg\min_q \epsilon_q.$$

*Stabilizing NMF* The NMF program is non-convex, and, empirically, individual replicates will not converge to the same optima. One solution therefore is to run multiple replicates of the NMF algorithm and cluster the resulting vectors. This approach raises the questions of how many clusters to use, and how to deal with stochasticity in the clustering algorithm itself. We address this issue through the notion of clustering stability (von Luxburg, 2010a).

The clustering stability approach is to generate $L$ replicas of k-cluster partitions $\{C_{kl} : l \in 1 \dots L\}$ and then compute the average dissimilarity between clusterings

$$\xi_k = \frac{2}{L(L-1)} \sum_{l=1}^{L} \sum_{l'=1}^{l} d(C_{kl}, C_{kl'}).$$

Then, the optimum number of clusters is

$$\hat{k} = \arg\min_k \xi_k.$$

A review of this approach is found in von Luxburg (2010b). Intuitively, archetype vectors that cluster together frequently over clustering replicates indicate the presence of a stable clustering. For $d$, we utilize the adjusted Rand Index - a simple dissimilarity measure between clusterings. Note that we expect to select slightly more than the $q$ components suggested by cross-validation, since archetype vectors which appear in one NMF replicate generally should appear in others. We then select the $q$ clusters with the most archetype vectors - the most stable NMF results - and take the median of each cluster to create a sparse representative archetype (Kotliar et al., 2019; Wu et al., 2016). We then find the according $H$ using Program 3. Experimental results for these cross-validation and stability selection approaches are given in Supplemental Section 7.

## 7 SUPPLEMENTAL EXPERIMENTS

The supplemental experiments show results on lower limit of detection, performance of our estimator for different regions and cell-classes, heirarchical clustering of connectivities, and stability and component analysis of our NMF results.

### *Setting detection threshold* $\tau$

We give results on the false detection rate at different limits of detection. These conclusively show that $10^{-6}$ is the good threshold for our normalized data.

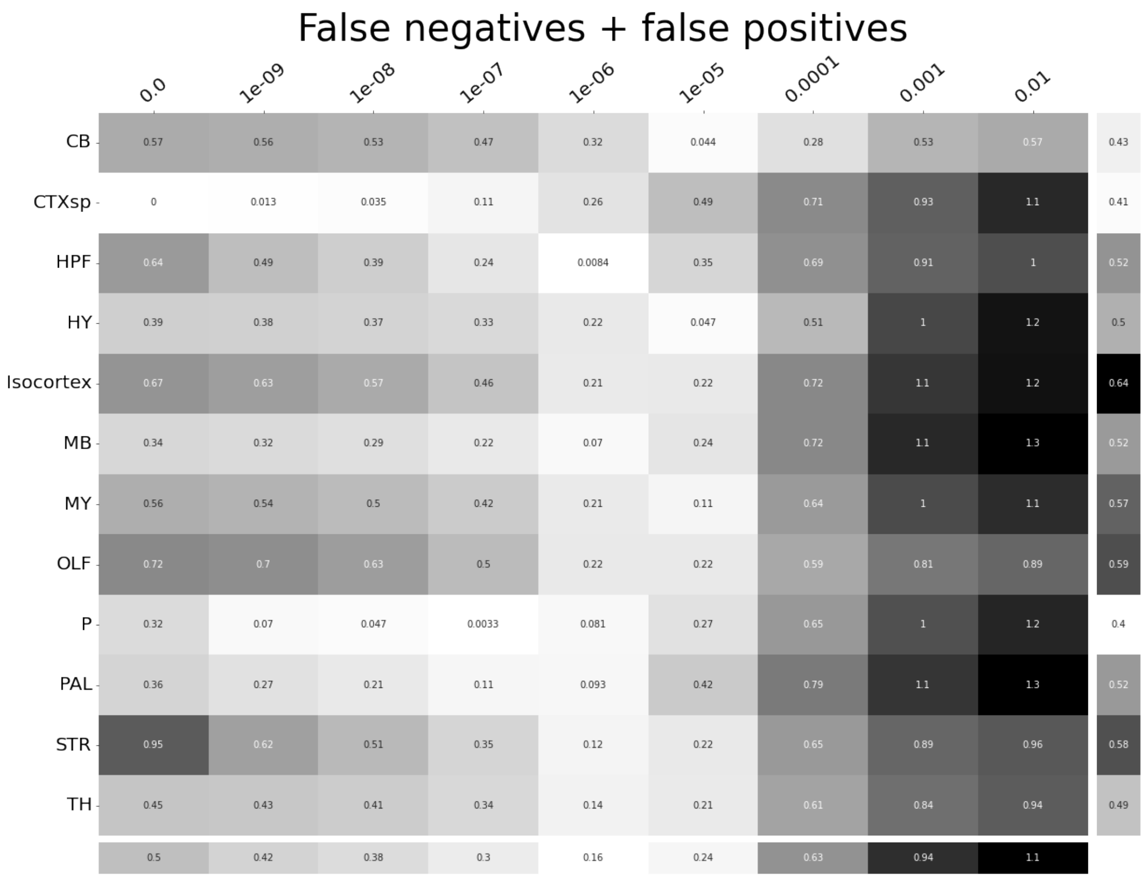


Figure 19: $\tau$ at different limits of detection in different major structures. $10^{-6}$ is the optimal detection threshold.

### *Loss subsets*

We report model accuracies for our *EL* model by neuron class and structure. These expand upon the results in Table 5 and give more specific information about the quality of our estimates. CTXsp is omitted due to the small evaluation set.

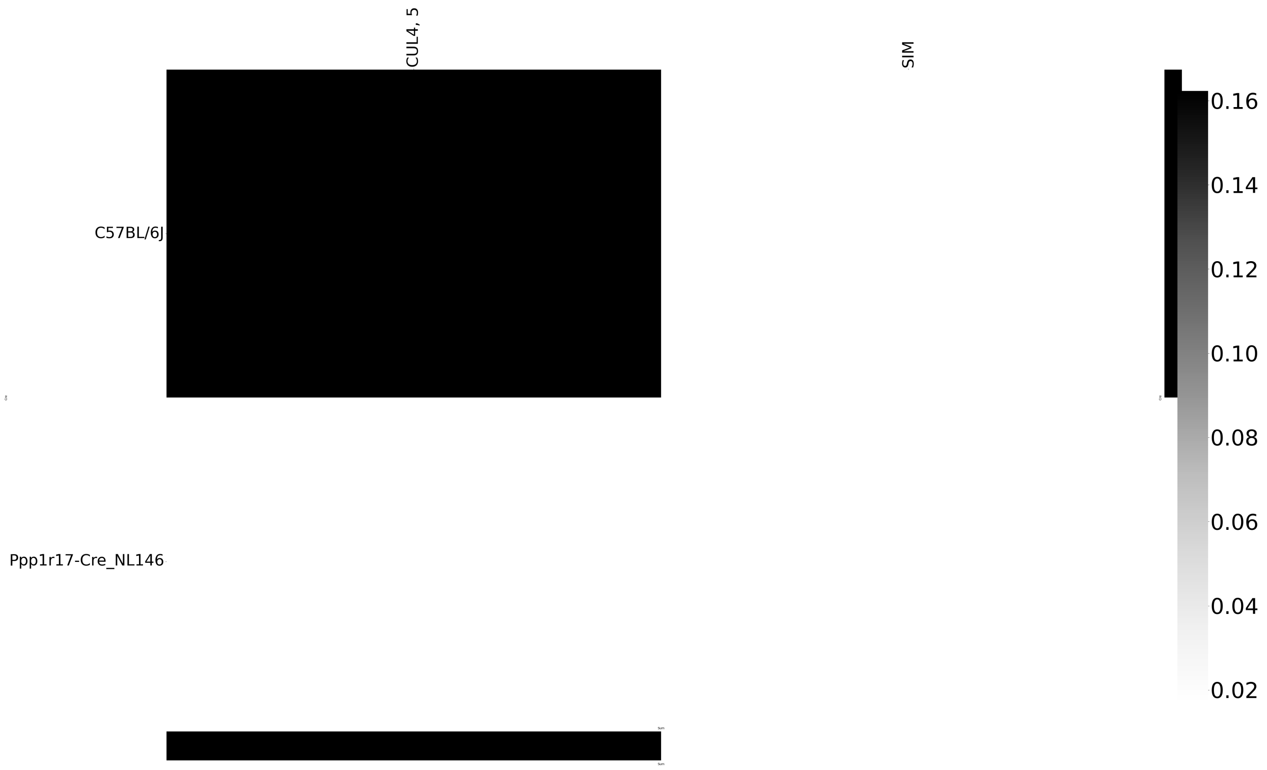


Figure 20: Weighted loss for Cre-leaf combinations in CB. Missing values are omitted. For example, this figure has one present and three missing values. Row and column averages are also plotted.

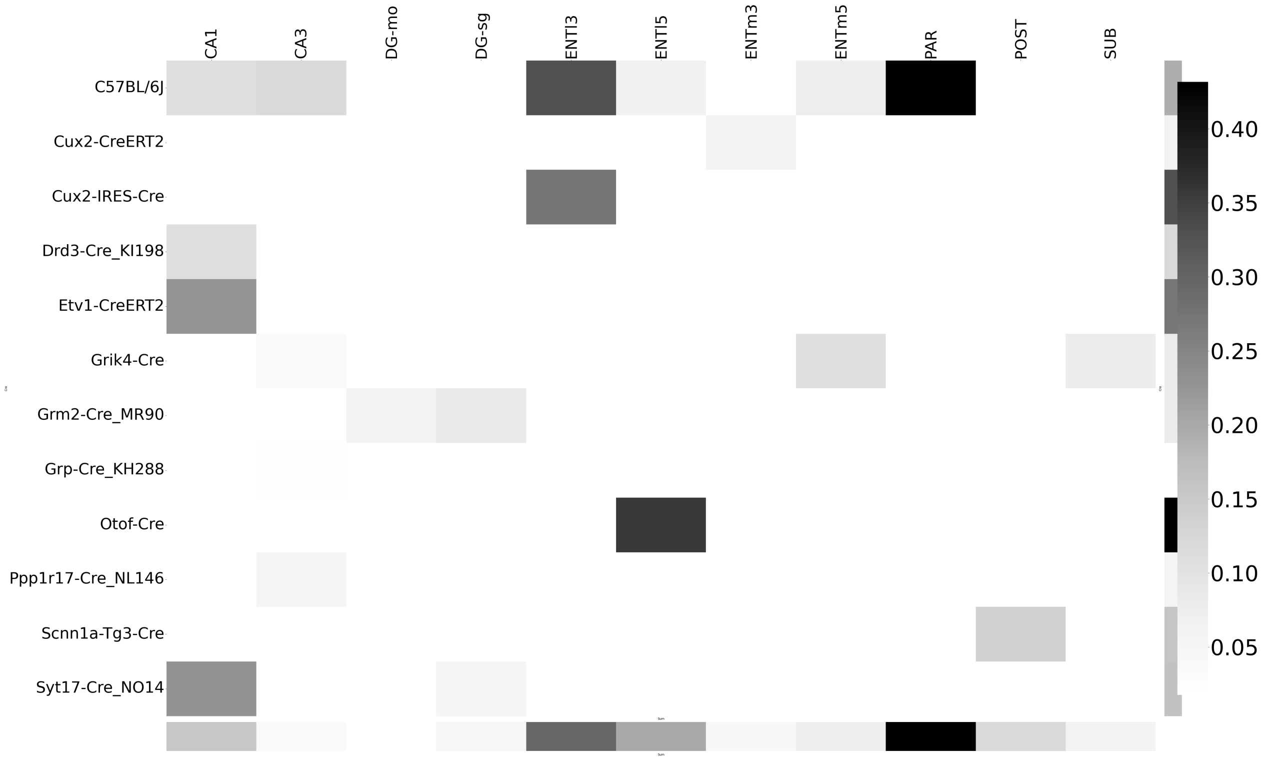


Figure 21: Weighted loss for Cre-leaf combinations in HPF. Missing values are omitted. Row and column averages are also plotted.

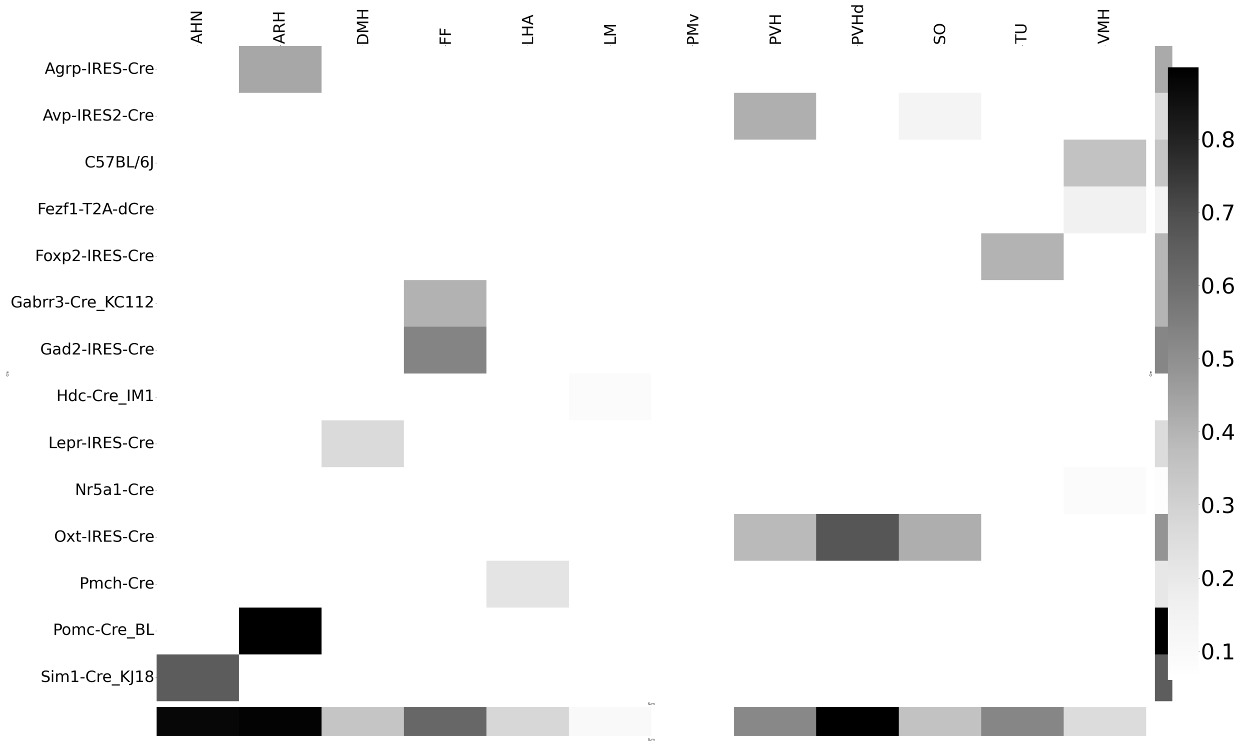


Figure 22: Weighted loss for Cre-leaf combinations in HY. Missing values are omitted. Row and column averages are also plotted.

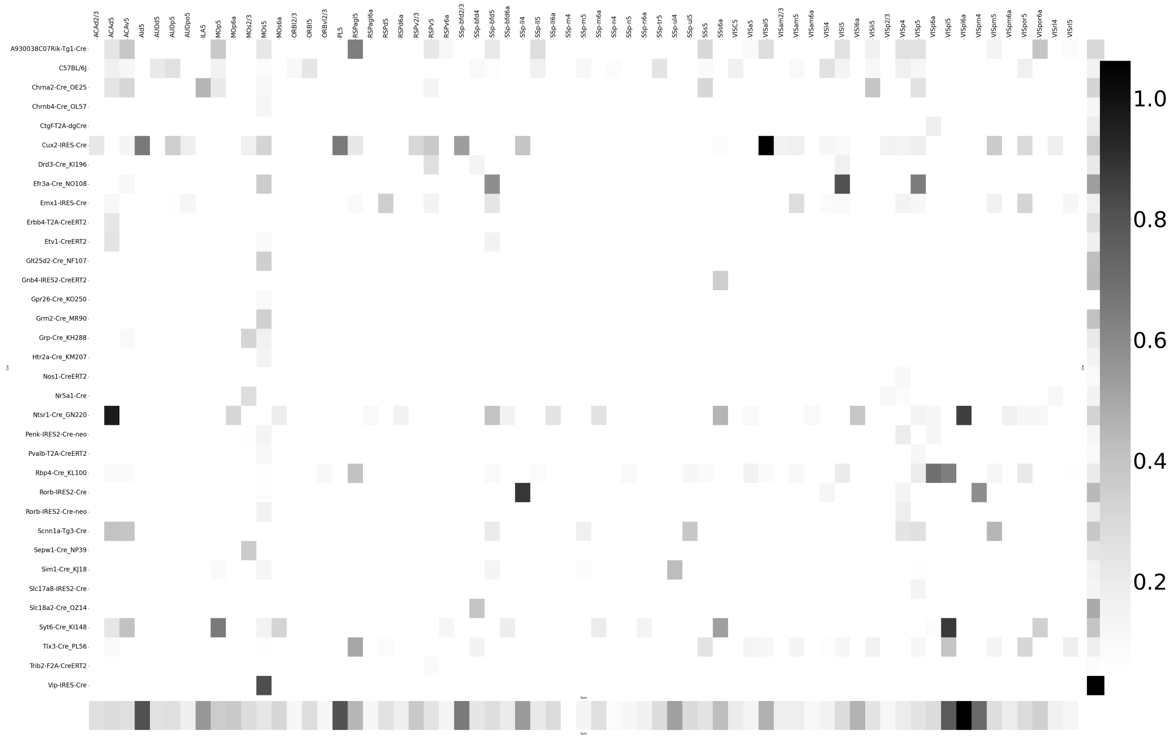


Figure 23: Weighted loss for Cre-leaf combinations in Isocortex. Missing values are omitted. Row and column averages are also plotted.

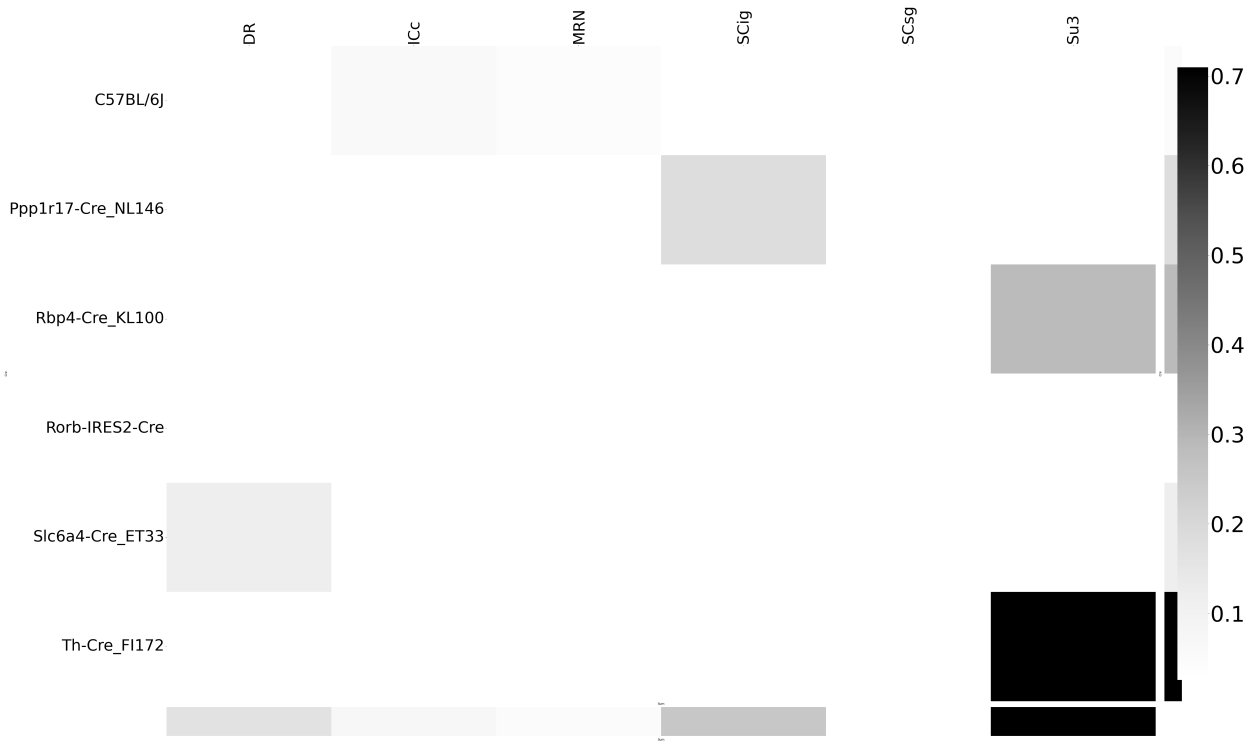


Figure 24: Weighted loss for Cre-leaf combinations in MB. Missing values are omitted. Row and column averages are also plotted.

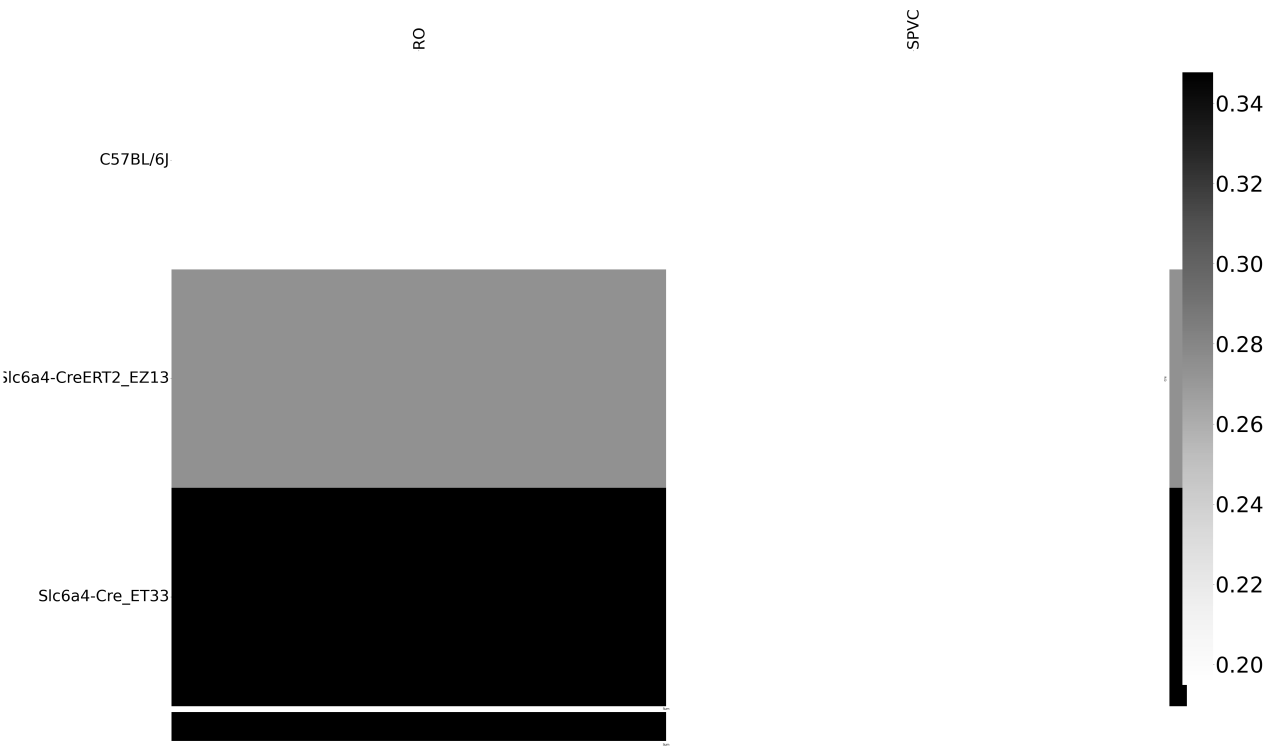


Figure 25: Weighted loss for Cre-leaf combinations in MY. Missing values are omitted. Row and column averages are also plotted.

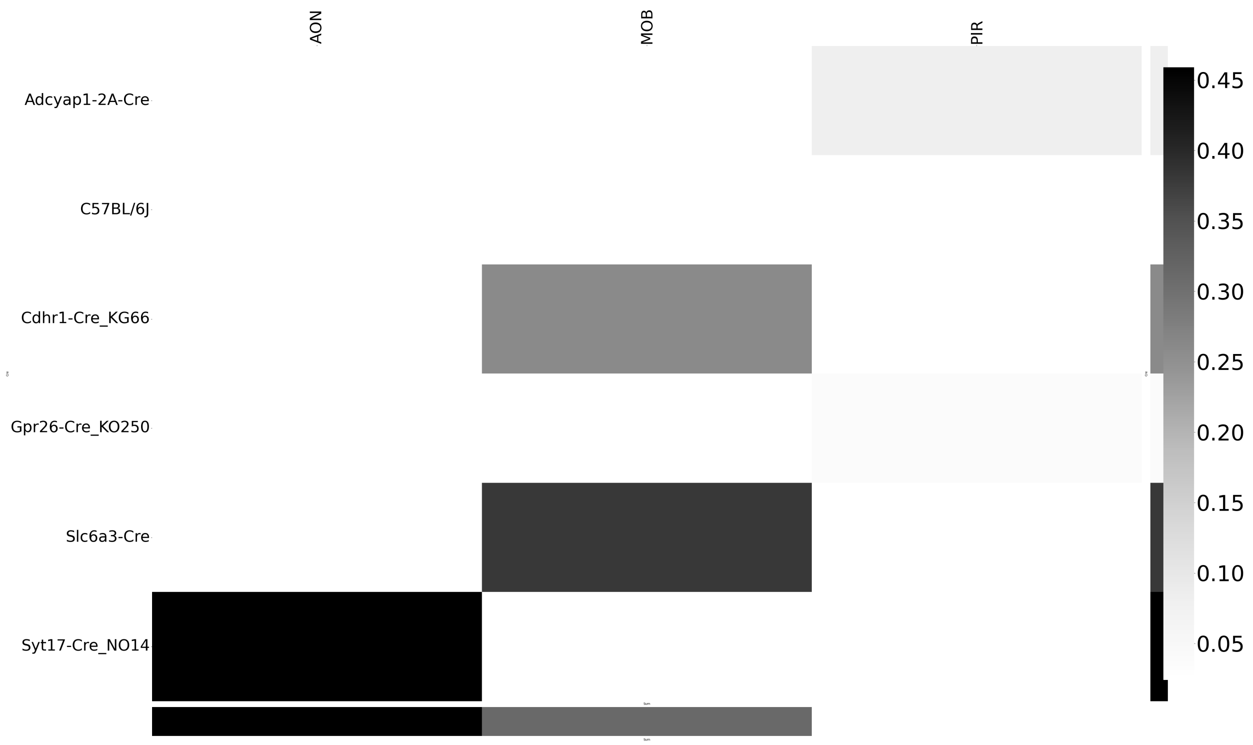


Figure 26: Weighted loss for Cre-leaf combinations in OLF. Missing values are omitted. Row and column averages are also plotted.

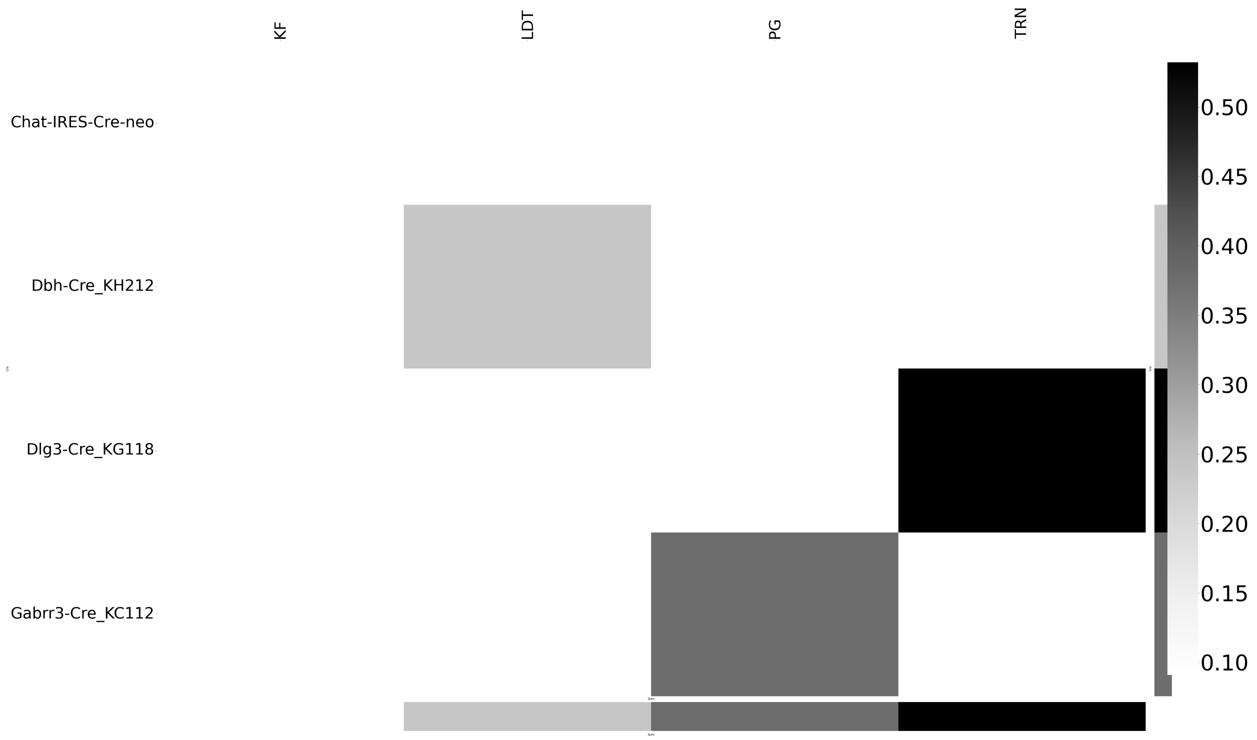


Figure 27: Weighted loss for Cre-leaf combinations in P. Missing values are omitted. Row and column averages are also plotted.

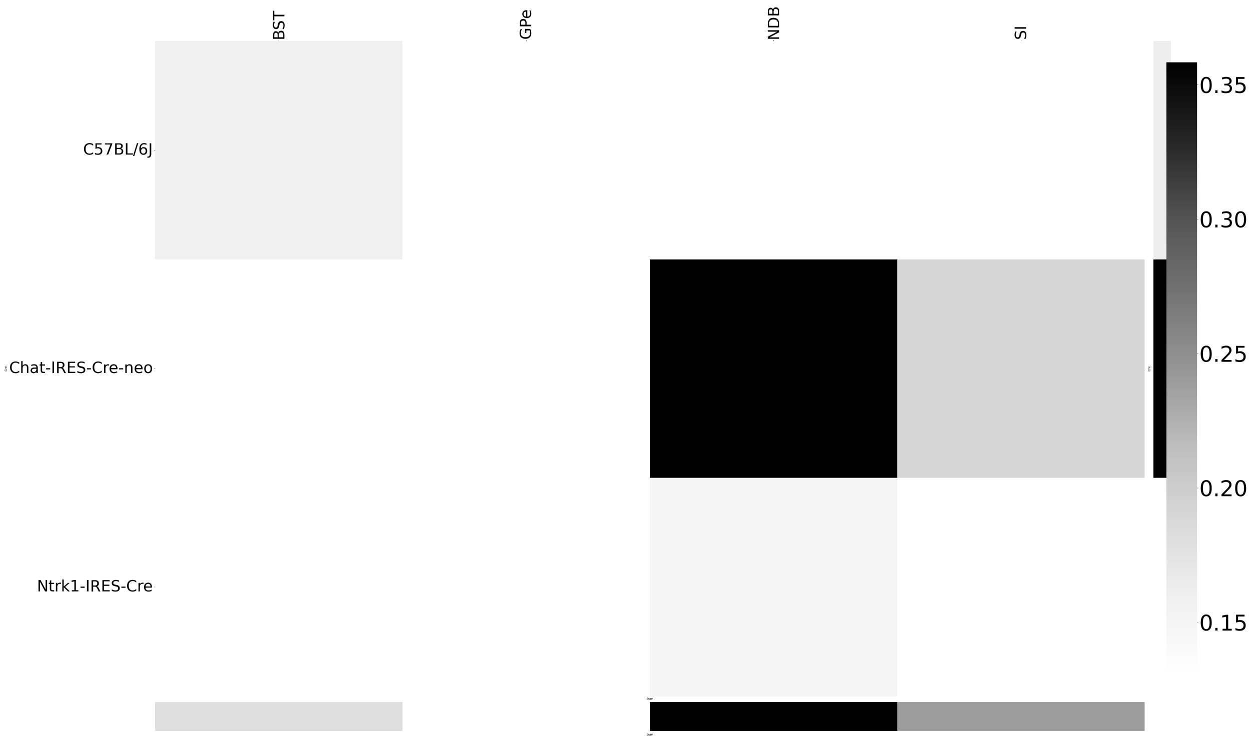


Figure 28: Weighted loss for Cre-leaf combinations in PAL. Missing values are omitted. Row and column averages are also plotted.

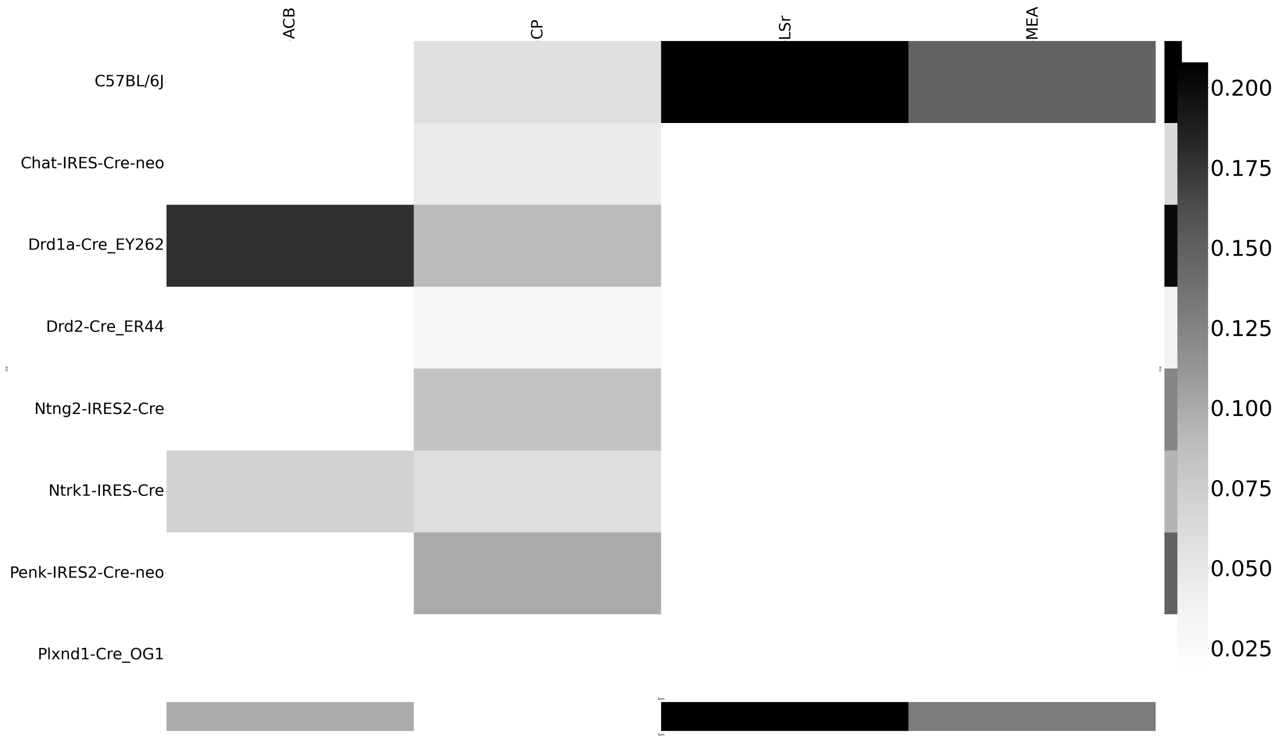


Figure 29: Weighted loss for Cre-leaf combinations in STR. Missing values are omitted. Row and column averages are also plotted.

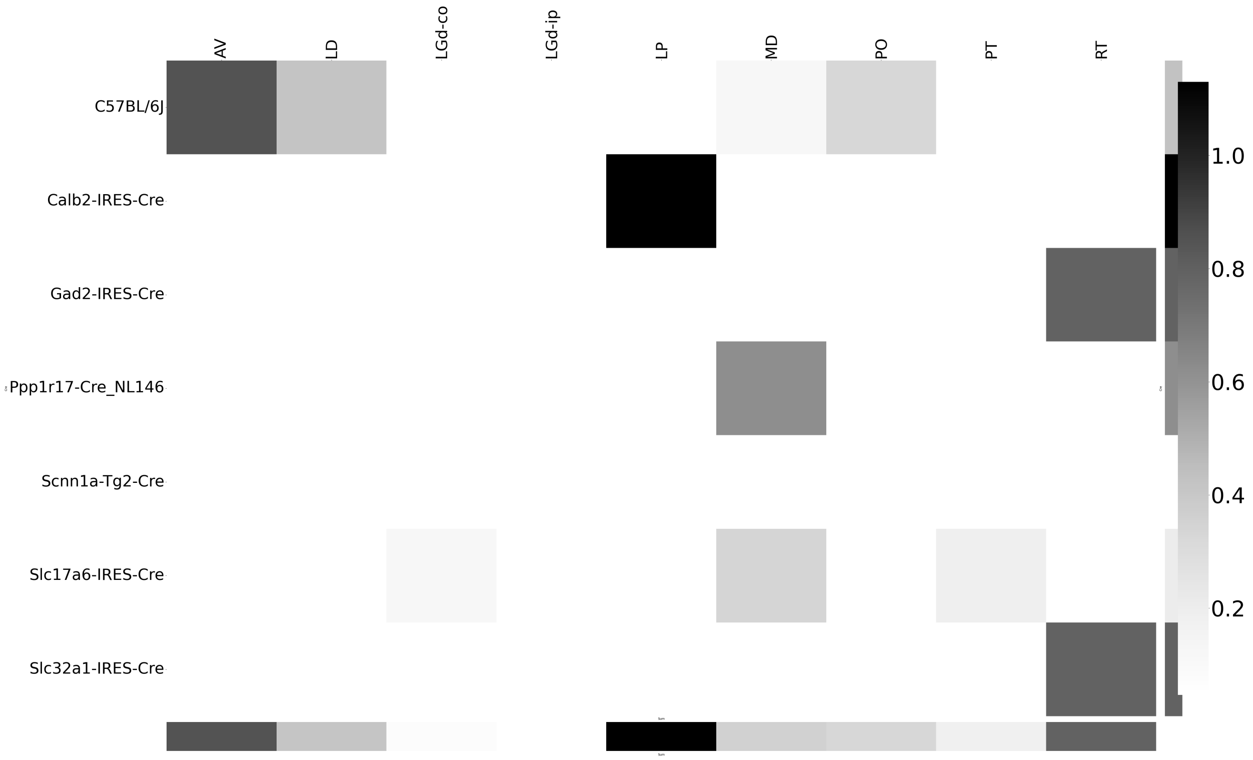


Figure 30: Weighted loss for Cre-leaf combinations in TH. Missing values are omitted. Row and column averages are also plotted.

### *Cell-type specificity*

We performed heirarchical clustering using the default method in Seaborn (Waskom, 2021) to investigate shared projection patterns across Cre-lines. That is, we used agglomerative clustering with Ward's criterion Hastie et al. (2009); Lalloué et al. (2013). This showed clustering of Ntsr1 projections to Thalamic nuceli.

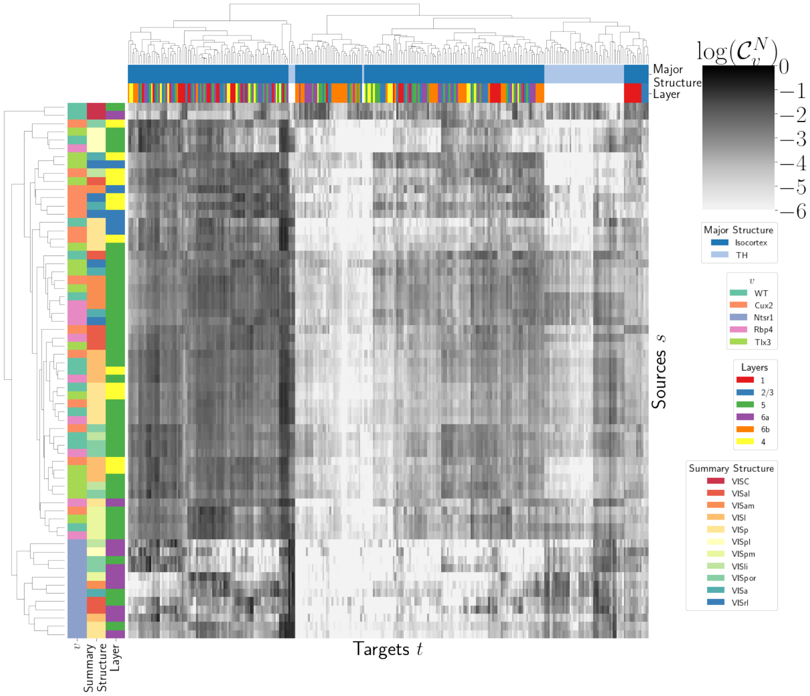


Figure 31: Hierarchical clustering of connectivity strengths from visual cortex cell-types to cortical and thalamic targets. Cre-line, summary structure, and layer are labelled on the sources. Major brain division and layer are labelled on the targets.

### *Matrix Factorization*

We give additional results on the generation of the archetypal connectome latent variables. These consist of cross-validation selection of $q$, the number of latent components, stability analysis, and visualization of the reconstructed wild-type connectivity.

*Cross-validation* We set $\alpha = 0.002$ and run Program 2 on $\mathscr{C}_{wt}$. We use a random mask with $p = .3$ to evaluate prediction accuracy of models trained on the unmasked data on the masked data. To account for stochasticity in the NMF algorithm, we run $R = 8$ replicates at each potential dimension $q$. The lowest mean test error was observed at $\hat{q} = 70$, indicating that even more components could be estimated. However, the low decrease in reconstruction error at higher values of $q$ and need for brevity in our figures motivated us to choose $q = 15$ for the purposes of display.

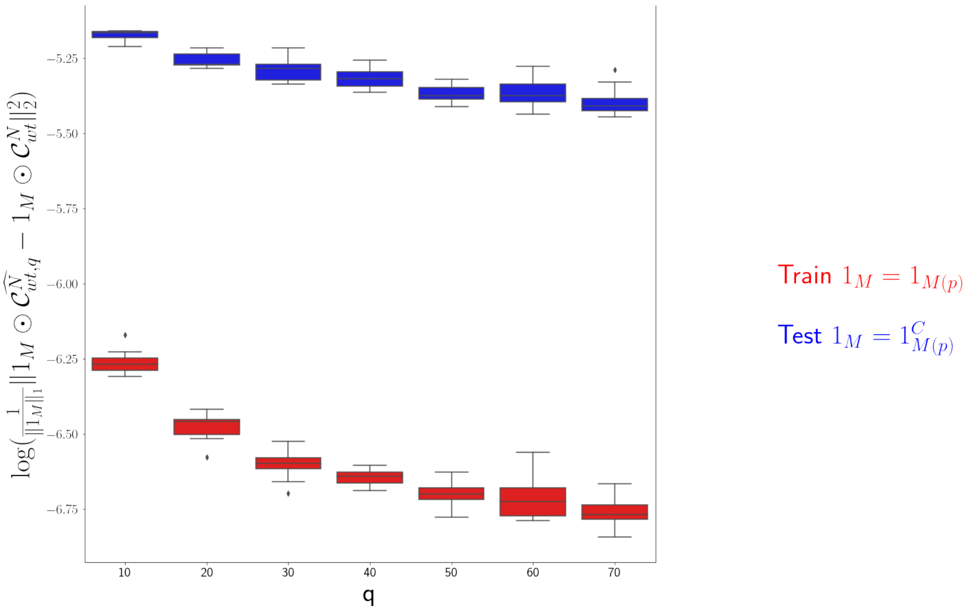


Figure 32: Train and test error using NMF decomposition.

*Stability* To address the instability of the NMF algorithm in identifying components, we k-means cluster components over $R = 10$ replicates with $k \in \{10, 15, 20, 25, 30\}$. Since the clustering is itself

unstable, we repeat the clustering 25 times and select the $k$ with the largest Rand index, a standard method of clustering stability (Meila, 2007; Rand, 1971).

| q | 10.000000 | 20.000000 | 30.000000 | 40.000000 | 50.000000 |
|---|---|---|---|---|---|
| Rand index | 0.772544 | 0.844981 | **0.932957** | 0.929827 | 0.885862 |

Since $k$-means is most stable at $k = 30$, we cluster the $qR = 150$ components into 30 clusters and select the 15 clusters appearing in the most replicates.

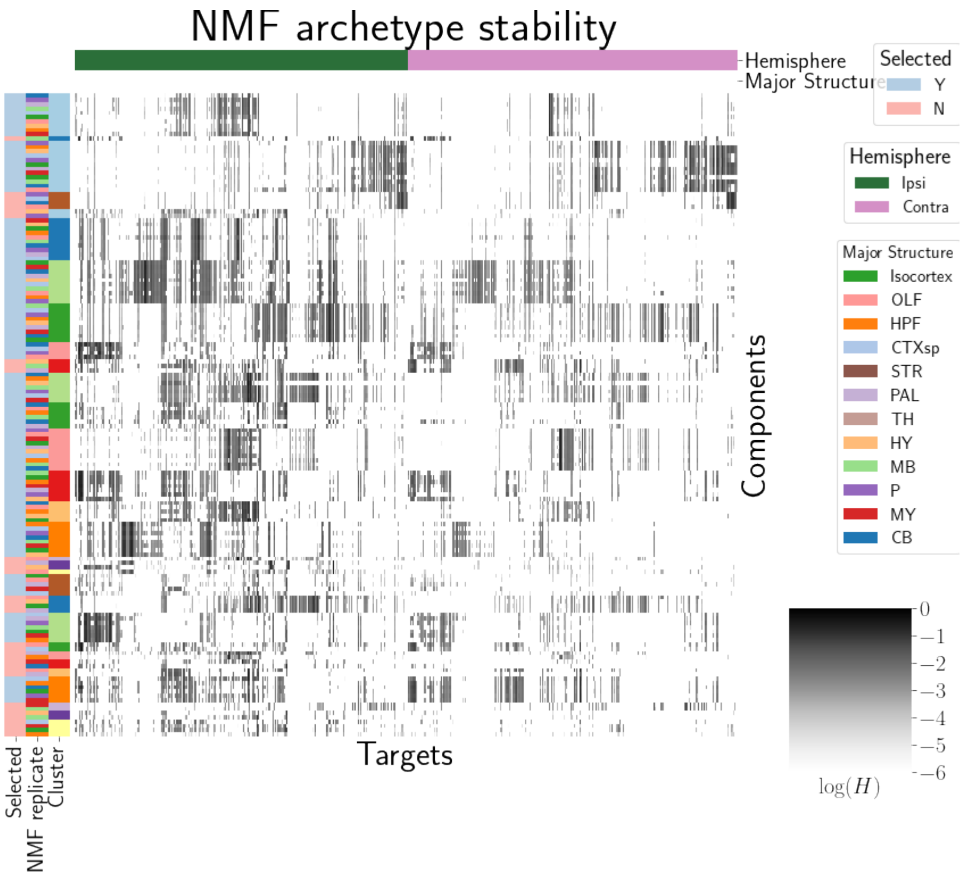


Figure 33: Stability of NMF results across replicates. Replicate and NMF component are shown on rows. Components that are in the top 15 are also indicated.

We plot the medians of these components in Figure 4a and in the main text. These are the connectivity archetypes. We then fit a non-negative least squares (the second step in the standard NMF optimization algorithm) to determine $W$ (Lee & Seung, 2000)

*Association with Cre-line* Finally, we show the association of our learned archetypes with projections from sources with injection centroids from the Ntsr1, Cux2, Rbp4, and Tlx3 Cre-lines. While we make no statistical claims on these associations, the distribution of cosine similarities of sources from each of the Cre-line lines shows an association of learned archetypes with Cre-line.

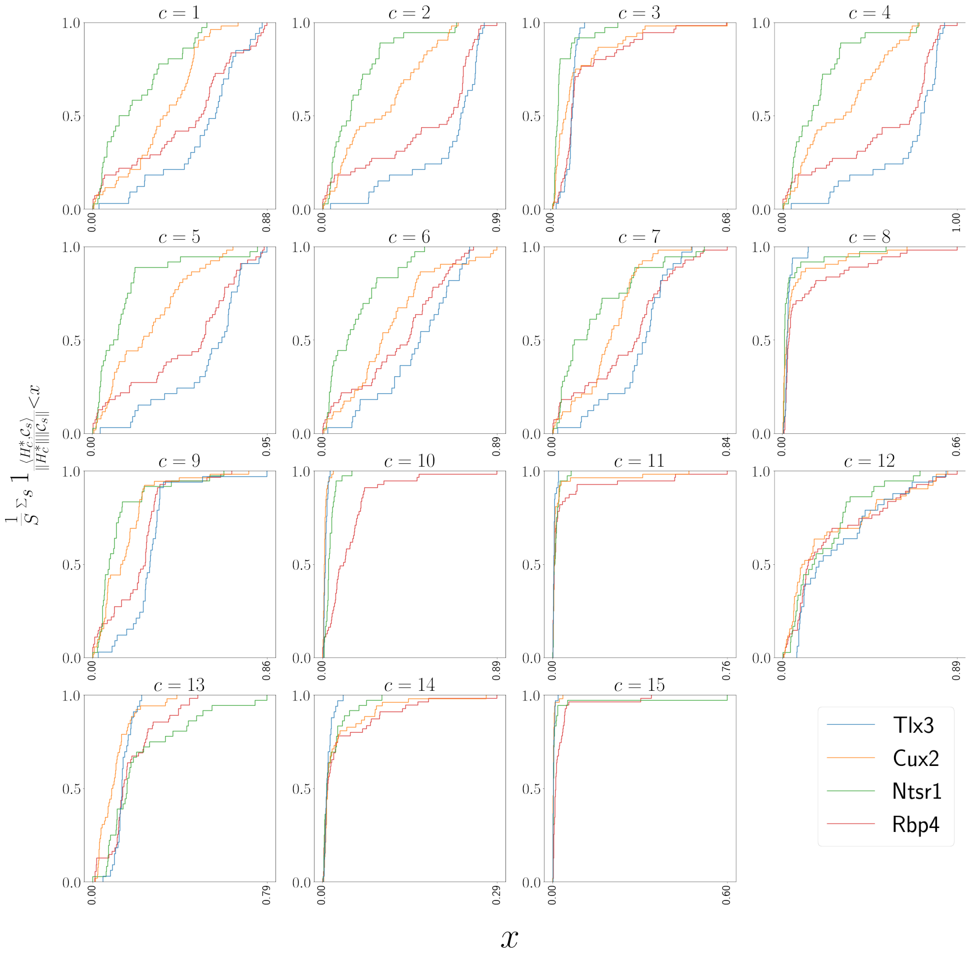


Figure 34: Empirical cumulative distributions of cosine similarities between source structures and connectivity components for four different Cre-lines.

# REFERENCES

Brunet, J.-P., Tamayo, P., Golub, T. R., & Mesirov, J. P. (2004). Metagenes and molecular pattern discovery using matrix factorization. *Proc. Natl. Acad. Sci. U. S. A.*, *101*(12), 4164–4169.

Cai, Z. (2001). Weighted Nadaraya-Watson regression estimation. *Stat. Probab. Lett.*, *51*(3), 307–318.

Chamberlin, N. L., Du, B., de Lacalle, S., & Saper, C. B. (1998). Recombinant adeno-associated virus vector: use for transgene expression and anterograde tract tracing in the CNS. *Brain Res.*, *793*(1-2), 169–175.

Daigle, T. L., Madisen, L., Hage, T. A., Valley, M. T., Knoblich, U., Larsen, R. S., … Zeng, H. (2018). A suite of transgenic driver and reporter mouse lines with enhanced Brain-Cell-Type targeting and functionality. *Cell*, *174*(2), 465–480.e22.

Devarajan, K. (2008). Nonnegative matrix factorization: an analytical and interpretive tool in computational biology. *PLoS Comput. Biol.*, *4*(7), e1000029.

Eilers, P. H. C., & Marx, B. D. (1996). Flexible smoothing with b-splines and penalties. *SSO Schweiz. Monatsschr. Zahnheilkd.*, *11*(2), 89–121.

Gămănuţ, R., Kennedy, H., Toroczkai, Z., Ercsey-Ravasz, M., Van Essen, D. C., Knoblauch, K., & Burkhalter, A. (2018). The mouse cortical connectome, characterized by an Ultra-Dense cortical graph, maintains specificity by distinct connectivity profiles. *Neuron*, *97*(3), 698–715.e10.

Gao, Y., Zhang, X., Wang, S., & Zou, G. (2016). Model averaging based on leave-subject-out cross-validation. *J. Econom.*, *192*(1), 139–151.

Groeneboom, P., & Jongbloed, G. (2018). Some developments in the theory of shape constrained inference. *Stat. Sci.*, *33*(4), 473–492.

Harris, J. A., Mihalas, S., Hirokawa, K. E., Whitesell, J. D., Choi, H., Bernard, A., … Zeng, H. (2019). Hierarchical organization of cortical and thalamic connectivity. *Nature*, *575*(7781), 195–202.

Harris, J. A., Oh, S. W., & Zeng, H. (2012). Adeno-associated viral vectors for anterograde axonal tracing with fluorescent proteins in nontransgenic and cre driver mice. *Curr. Protoc. Neurosci.*, *Chapter 1*, Unit 1.20.1–18.

Harris, K. D., Mihalas, S., & Shea-Brown, E. (2016). Nonnegative spline regression of incomplete tracing data reveals high resolution neural connectivity.

Hastie, T., Tibshirani, R., & Friedman, J. (2009). *The elements of statistical learning*. Springer New York.

Huang, K. W., Ochandarena, N. E., Philson, A. C., Hyun, M., Birnbaum, J. E., Cicconet, M., & Sabatini, B. L. (2019). Molecular and anatomical organization of the dorsal raphe nucleus. *Elife*, *8*.

Jackson, K. L., Dayton, R. D., Deverman, B. E., & Klein, R. L. (2016). Better targeting, better efficiency for Wide-Scale neuronal transduction with the synapsin promoter and AAV-PHP.B. *Front. Mol. Neurosci.*, *9*, 116.

Jeong, M., Kim, Y., Kim, J., Ferrante, D. D., Mitra, P. P., Osten, P., & Kim, D. (2016). Comparative three-dimensional connectome map of motor cortical projections in the mouse brain. *Sci. Rep.*, *6*, 20072.

Knox, J. E., Harris, K. D., Graddis, N., Whitesell, J. D., Zeng, H., Harris, J. A., … Mihalas, S. (2019). High-resolution data-driven model of the mouse connectome. *Netw Neurosci*, *3*(1), 217–236.

Kotliar, D., Veres, A., Nagy, M. A., Tabrizi, S., Hodis, E., Melton, D. A., & Sabeti, P. C. (2019). Identifying gene expression programs of cell-type identity and cellular activity with single-cell RNA-Seq. *Elife*, *8*.

Kuan, L., Li, Y., Lau, C., Feng, D., Bernard, A., Sunkin, S. M., … Ng, L. (2015). Neuroinformatics of the allen mouse brain connectivity atlas. *Methods*, *73*, 4–17.

Kügler, S., Kilic, E., & Bähr, M. (2003). Human synapsin 1 gene promoter confers highly neuron-specific long-term transgene expression from an adenoviral vector in the adult rat brain depending on the transduced area. *Gene Ther.*, *10*(4), 337–347.

Lalloué, B., Monnez, J.-M., Padilla, C., Kihal, W., Le Meur, N., Zmirou-Navier, D., & Deguen, S. (2013). A statistical procedure to create a neighborhood socioeconomic index for health inequalities analysis. *Int. J. Equity Health*, *12*, 21.

Lee, D., & Seung, H. S. (2000). Algorithms for non-negative matrix factorization. *Adv. Neural Inf. Process. Syst.*, *13*.

Li, X., Yu, B., Sun, Q., Zhang, Y., Ren, M., Zhang, X., … Qiu, Z. (2018). Generation of a whole-brain atlas for the cholinergic system and mesoscopic projectome analysis of basal forebrain cholinergic neurons. *Proc. Natl. Acad. Sci. U. S. A.*, *115*(2), 415–420.

Llano, D. A., & Sherman, S. M. (2008). Evidence for nonreciprocal organization of the mouse auditory thalamocortical-corticothalamic projection systems. *J. Comp. Neurol.*, *507*(2), 1209–1227.

Lotfollahi, M., Naghipourfar, M., Theis, F. J., & Alexander Wolf, F. (2019). Conditional out-of-sample generation for unpaired data using trVAE.

Meila, M. (2007). Comparing clusterings—an information based distance. *J. Multivar. Anal.*, *98*(5), 873–895.

Mohammadi, S., Ravindra, V., Gleich, D. F., & Grama, A. (2018). A geometric approach to characterize the functional identity of single cells. *Nat. Commun.*, *9*(1), 1516.

Muzerelle, A., Scotto-Lomassese, S., Bernard, J. F., Soiza-Reilly, M., & Gaspar, P. (2016). Conditional anterograde tracing reveals distinct targeting of individual serotonin cell groups (B5-B9) to the forebrain and brainstem. *Brain Struct. Funct.*, *221*(1), 535–561.

Oh, S. W., Harris, J. A., Ng, L., Winslow, B., Cain, N., Mihalas, S., … Zeng, H. (2014). A mesoscale connectome of the mouse brain. *Nature*, *508*(7495), 207–214.

Perry, P. O. (2009). Cross-Validation for unsupervised learning.

Rand, W. M. (1971). Objective criteria for the evaluation of clustering methods. *J. Am. Stat. Assoc.*, *66*(336), 846–850.

Ren, J., Friedmann, D., Xiong, J., Liu, C. D., Ferguson, B. R., Weerakkody, T., … Luo, L. (2018). Anatomically defined and functionally distinct dorsal raphe serotonin sub-systems. *Cell*, *175*(2), 472–487.e20.

Ren, J., Isakova, A., Friedmann, D., Zeng, J., Grutzner, S. M., Pun, A., … Luo, L. (2019). Single-cell transcriptomes and whole-brain projections of serotonin neurons in the mouse dorsal and median raphe nuclei. *Elife*, *8*.

Salha, R. B., & El Shekh Ahmed, H. I. (2015). Reweighted Nadaraya-Watson estimator of the regression mean. *International Journal of Statistics and Probability*, *4*(1).

Saul, L. K., & Roweis, S. T. (2003). Think globally, fit locally: Unsupervised learning of low dimensional manifolds. *J. Mach. Learn. Res.*, *4*(Jun), 119–155.

Saunders, A., Johnson, C. A., & Sabatini, B. L. (2012). Novel recombinant adeno-associated viruses for cre activated and inactivated transgene expression in neurons. *Front. Neural Circuits*, *6*, 47.

Servén, D., & Brummitt, C. (2018). *pygam: Generalized additive models in python.* doi: 10.5281/zenodo.1208723

von Luxburg, U. (2010a). Clustering stability: An overview.

von Luxburg, U. (2010b). Clustering stability: An overview.

Wang, Q., Ding, S.-L., Li, Y., Royall, J., Feng, D., Lesnar, P., … Ng, L. (2020). The allen mouse brain common coordinate framework: A 3D reference atlas. *Cell*, *181*(4), 936–953.e20.

Waskom, M. L. (2021). seaborn: statistical data visualization. *Journal of Open Source Software*, *6*(60), 3021. Retrieved from https://doi.org/10.21105/joss.03021 doi: 10.21105/joss.03021

Watson, C., Paxinos, G., & Puelles, L. (2012). The mouse nervous system..

Wu, S., Joseph, A., Hammonds, A. S., Celniker, S. E., Yu, B., & Frise, E. (2016). Stability-driven nonnegative matrix factorization to interpret spatial gene expression and build local gene networks. *Proc. Natl. Acad. Sci. U. S. A.*, *113*(16), 4290–4295.

Zaborszky, L., Csordas, A., Mosca, K., Kim, J., Gielow, M. R., Vadasz, C., & Nadasdy, Z. (2015). Neurons in the basal forebrain project to the cortex in a complex topographic organization that reflects corticocortical connectivity patterns: an experimental study based on retrograde tracing and 3D reconstruction. *Cereb. Cortex*, *25*(1), 118–137.
